# Comprehensive analysis of the flowering genes in Chinese cabbage and examination of evolutionary pattern of CO-like genes in plant kingdom

**DOI:** 10.1038/srep14631

**Published:** 2015-09-29

**Authors:** Xiaoming Song, Weike Duan, Zhinan Huang, Gaofeng Liu, Peng Wu, Tongkun Liu, Ying Li, Xilin Hou

**Affiliations:** 1 State Key Laboratory of Crop Genetics and Germplasm Enhancement/Key Laboratory of Biology and Germplasm Enhancement of Horticultural Crops in East China, Ministry of Agriculture, Nanjing Agricultural University, Nanjing 210095, China; 2 Center of Genomics and Computational Biology, College of Life Sciences, North China University of Science and Technology, Tangshan, Hebei 063000, China

## Abstract

In plants, flowering is the most important transition from vegetative to reproductive growth. The flowering patterns of monocots and eudicots are distinctly different, but few studies have described the evolutionary patterns of the flowering genes in them. In this study, we analysed the evolutionary pattern, duplication and expression level of these genes. The main results were as follows: (i) characterization of flowering genes in monocots and eudicots, including the identification of family-specific, orthologous and collinear genes; (ii) full characterization of CONSTANS-like genes in *Brassica rapa* (*BraCOL* genes), the key flowering genes; (iii) exploration of the evolution of *COL* genes in plant kingdom and construction of the evolutionary pattern of *COL* genes; (iv) comparative analysis of *CO* and *FT* genes between *Brassicaceae* and *Grass*, which identified several family-specific amino acids, and revealed that CO and FT protein structures were similar in *B. rapa* and *Arabidopsis* but different in rice; and (v) expression analysis of photoperiod pathway-related genes in *B. rapa* under different photoperiod treatments by RT-qPCR. This analysis will provide resources for understanding the flowering mechanisms and evolutionary pattern of *COL* genes. In addition, this genome-wide comparative study of *COL* genes may also provide clues for evolution of other flowering genes.

In plants, successful sexual reproduction and seed development depend on flowering at the appropriate time^1^. The transition from vegetative to reproductive growth is one of the most important developmental switches in the plant life cycle^2^,^3^. A diverse range of endogenous and environmental signals regulates this transition, and these signals are integrated into a single decision—to flower or not. The interactions between proteins that transduce and integrate these signals promote or inhibit the transition to flowering^4^,^5^. In *Arabidopsis*, most key flowering genes have been identified and functionally characterized^1^,^6^,^7^. Genetic and molecular studies have revealed that many genes are involved and can be assigned to distinct regulatory pathways, including the vernalization, photoperiod, gibberellin, autonomous, ambient temperature and aging pathways^1^,^6^. Interestingly, it has been discovered that many homologous genes of *Arabidopsis* regulate flowering in other species^8^,^9^.

The identified flowering genes are involved in a network of six regulatory pathways (Supplementary Fig. S1). These pathways converge to regulate a set of ‘floral integrator’ genes that integrate the outputs of the various pathways and directly activate the identity genes of the floral meristem^1^,^3^,^10^. These integrator genes include *SUPPRESSOR OF OVEREXPRESSION OF CONSTANS 1* (*SOC1*), *FLOWERING LOCUS T* (*FT*), *TWIN SISTER OF FT* (*TSF*), *FLOWERING LOCUS C* (*FLC*) and *LEAFY* (*LFY*)^6^. *TSF* is the closest homolog of *FT* in Arabidopsis, and their mRNA levels present similar patterns of diurnal oscillation and response to photoperiod. *TSF* promotes flowering largely redundantly with *FT* but makes a distinct contribution under SD conditions^11^. The expression of these floral promoters leads to further up-regulation of *LFY* and other floral-identity genes, such as *APETALA 1* (*AP1*), and then promotes flowering^12^,^13^. Changing the seasonal timing of flowering is a major goal of plant breeding, which may allow the production of novel varieties adapted to local climatic and environmental conditions^5^. The day length can control several plant processes, including flowering time, formation of bud dormancy and production of storage organs. *Arabidopsis*, a model eudicot, is a facultative long-day (LD) plant because its flowering is promoted by long days and delayed by short days, whereas rice is a typical short-day (SD) monocot. In all seed crops, floral transition is a key developmental switch that determines the production of dry matter^3^,^5^.

The photoperiod regulates flowering mainly by the *CONSTANS* (*CO*) and *FT* genes^14^,^15^,^16^,^17^,^18^. *CO* is a phloem-specific transcription activator of *FT* that promotes flowering by up-regulating the expression of *FT* and *SOC1* genes^19^,^20^,^21^,^22^,^23^,^24^. These genes are up-regulated in LD plants, resulting in rapid flowering. The CO-like genes delay flowering, while FT-like genes promote flowering in SD plants^1^,^25^. Previous analyses have demonstrated that the timing of the formation of the *FLAVIN-BINDING, KELCH REPEAT, F-BOX 1* (*FKF1*) and *GIGANTEA* (*GI*) gene complex determines the timing of daytime *CO* gene expression in LD plants^26^. The role of the *FKF1-GI* complex is to remove *CYCLING DOF FACTORs* (*CDFs*), which are transcriptional repressors of *CO*^27^. At the posttranscriptional level, *CO* is degraded in the dark by *CONSTITUTIVE PHOTOMORPHOGENIC 1* (*COP1*) and destabilized in the morning by phytochrome B (PHYB). However, *CO* is promoted by CRY and phytochrome A (PHYA)^28^,^29^. Therefore, the stability of *CO* based on light and the circadian clock is the core of day-length-sensing mechanisms^21^,^28^,^29^. These regulatory mechanisms ensure that *CO* activates *FT* transcription only during long days. The expression and translation of *FT* occur in the leaves, and the translated protein translocates to the meristem, where it activates floral development. The FT protein is a major florigen and is synthesized in the leaf vasculature^18^,^20^,^30^,^31^. The FT protein then travels to the shoot apical meristem to initiate the expression of floral-identity genes^32^,^33^. Previous analyses have shown that the *CO*/*FT* module is highly conserved in most angiosperms^34^. *CO* and *FT* orthologs are found in LD and SD plants. In rice, *Hd1* and *Hd3a* are the orthologs of *Arabidopsis CO* and *FT,* respectively^25^,^35^. In addition, *RICE FLOWERING LOCUS T 1* (*RFT1*) is the closest homologue of Hd3a and is thus also an ortholog of *Arabidopsis CO*^36^. The *CO*/*FT* module plays an important role in flowering-time regulation^37^,^38^. The functions of the key genes in the pathways controlling photoperiodic flowering are the same in both *Arabidopsis* and rice. However, the regulation of flowering by *Hd1* is different under long-day conditions. Under LD conditions, *CO* promotes *FT* expression in *Arabidopsis*, but *Hd1* represses *Hd3a* in rice. Under SD conditions, *Hd1* promotes flowering and up-regulates *Hd3a*, which differs from the function of *Arabidopsis CO*^25^,^35^,^39^. The time-keeping mechanism with a periodicity of 24 hours is known as the circadian clock, which confers diurnal patterns on gene expression. It has three interlocked feed-back loops: the central, morning and evening loops. The central loop contains the transcription factors *LATE ELONGATED HYPOCOTYL* (*LHY*) and *CIRCADIAN CLOCK ASSOCIATED 1* (*CCA1*), which repress the transcription of *TIMING OF CAB 1* (*TOC1*). TOC1 encodes a pseudo-response regulator and activates the transcription of *LHY* and *CCA1*. Similar loops exist in the morning and evening, and these include *PRR7/PRR9* and *GI*. The circadian clock ensures that *CO* transcription peaks late in the day, and *GI* enhances this peak under LD conditions^6^.

Until now, several studies have investigated the *CO* and *FT* genes in plants, and their evolution in different species has been investigated^17^,^37^,^40^,^41^,^42^,^43^,^44^,^45^,^46^,^47^,^48^,^49^,^50^,^51^,^52^,^53^,^54^. However, there remain several questions that need to be answered. For example, the event/protein that changes *Hd1* from a repressor of rice flowering under LD conditions to an activator of flowering under SD conditions has not yet been identified. It is also unclear why *Arabidopsis CO* and rice *Hd1* exert opposite effects on flowering under LD conditions. Owing to the increasing number of sequenced genomes from different species, it is currently possible and necessary to systematically analyse these genes. Chinese cabbage is one of the most important vegetables worldwide. Given its significant economic value and close relationship to *Arabidopsis*, the Chinese cabbage (Chiifu-401-42) genome has been sequenced and assembled^55^. Chinese cabbage is a LD biennial plant that flowers in the spring after undergoing vernalization in the winter. However, despite the important role of flowering genes in growth regulation, few studies have systematically investigated these genes in Chinese cabbage. Thus, the investigation of flowering-related genes in the whole Chinese cabbage genome is timely. In this study, we comprehensively identified the flowering genes of this species through comparative genomic analysis. Seventy-three common gene families were identified among 11 species using OrthoMCL^56^. The evolutionary divergence of the genetic factors underlying the differences in morphological and physiological features between monocots and eudicots is of great interest. Grasses, including the most important cereal crops, are typically monocots. However, few studies have comprehensively compared the evolutionary history of flowering genes at the whole-genome level between these two groups. In this study, we compared the gene number, gene duplication and class-specific genes involved in the flowering time of one basal angiosperm, four grasses and six eudicots. Furthermore, we also determined the evolutionary pattern and history of the *COL* genes in the plant kingdom.

## Results and Discussion

### Characterization of flowering genes in monocots and eudicots

**Figure 1:**
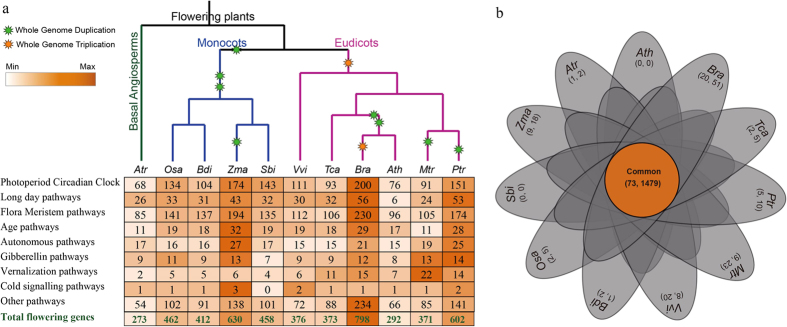
The comparative analysis of flowering-related genes in angiosperms. (**a**) The number of genes in each flowering-related pathway in six eudicots, four monocots and one basal angiosperm. Information regarding genome duplication or triplication was obtained from the Plant Genome Duplication Database (PGDD)^70^. (**b**) The Venn diagram shows the number of common and specific gene families and genes in 11 angiosperms. The first number in the brackets represents the number of gene families, and the second number represents the number of genes. The abbreviations represent the species as follows: *Ath, Arabidopsis thaliana*; *Bra, Brassica rapa*; *Tca, Theobroma cacao*; *Ptr, Populus trichocarpa*; *Mtr, Medicago truncatula*; *Vvi, Vitis vinifera*; *Bdi, Brachypodium distachyon*; *Osa, Oryza sativa*; *Sbi, Sorghum bicolor*; *Zma, Zea mays*; *Atr, Amborella trichopoda*.

We systematically collected all flowering-related genes of *Arabidopsis* according to previous reports^1^,^3^,^6^. Based on previously reported methods^57^,^58^, the homogeneous candidate flowering genes between *Arabidopsis* and other species were identified by BLAST (E-value <1 × 10^−10^, Identity>40%) (Fig. 1a, Supplementary Table S1). There were more flowering genes in *B. rapa* (798) than in the other ten species, and *A. trichopoda* had the least flowering genes (273) (Supplementary Table S2). This result may indicate that *B. rapa* underwent genome triplication, whereas *A. trichopoda* did not. In monocots, we noted that *Z. mays* had more flowering genes (630) compared with the other three monocots because it had undergone an additional whole-genome duplication (WGD) event. In eudicots, although *B. rapa* had the most flowering genes, only four pathways were found to have more genes than those found in the other five eudicots.

We identified common and specific flowering genes in these 11 angiosperms according to a previous report^58^. Seventy-three subfamilies were common to the 11 species, and these contained 1479 genes (Fig. 1b). *B. rapa* contained the highest number of specific subfamilies (20), followed by *M. truncatula* (9) and *Z. mays* (9). The monocots contained 68 specific subfamilies (319 genes), and the eudicots had eight specific subfamilies (72 genes). However, *A. trichopoda*, a basal angiosperm species, only contained one specific subfamily, which includes two genes (Supplementary Fig. S2a). To further analyse species-specific genes in eudicots and monocots, four monocots and six eudicots were analysed. The results revealed that *B. distachyon*, rice and *Z. mays* contained several specific genes, whereas no specific gene was identified in *S. bicolor* (Supplementary Fig. S2b). Similarly, we did not find any specific genes in *Arabidopsis* (Supplementary Fig. S2c).

To analyse the duplication events of flowering genes, we identified the duplicated type for each flowering gene and found that *B. rapa* and *P. trichocarpa* contained more flowering genes that had undergone a WGD or segmental duplication, namely 78.20% and 76.08%, respectively (Supplementary Table S2). Interestingly, we noted that the percentage of flowering genes that had undergone a WGD or segmental duplication in all the species (except *A. trichopoda*) was greater than that obtained at the whole-genome level (Supplementary Table S3). This phenomenon indicated that the flowering genes expanded during WGD events. Because no genome duplication event occurred during the evolution of *A. trichopoda*, it contained very few flowering genes that had undergone a WGD or segmental duplication (0.73%), and most of its flowering genes were dispersed (88.28%).

Detection of collinear orthologs is very important for understanding gene evolution in different species. The ratio of collinear ortholog pairs to all ortholog pairs was calculated (Table 1, Supplementary Table S4). This ratio can reveal how gene orders are conserved or how frequently chromosomes are rearranged between species^59^. The results showed that the gene order is better conserved within monocots and eudicots than between monocots and eudicots. However, we also found several exceptions, such as *B. rapa* and *M. truncatula*. Although these species are eudicots, the ratios of collinear ortholog pairs to all ortholog pairs obtained for their flowering genes (7.09%) and their whole genomes (4.84%) were very low, which demonstrated that the genes were rearranged between these two species. In addition, we obtained a markedly higher ratio between *S. bicolor* and *V. vinifera* for the flowering genes (48.66%) than for the whole genome (7.01%). This phenomenon indicated that the order of the flowering genes was better conserved than the order of the whole genome between these two species. In contrast, a markedly higher ratio was found between *M. truncatula* and rice for the whole genome (47.58%) than for the flowering genes (2.43%), which suggested that the flowering genes were rearranged between these two species. *A. trichopoda* was found to exhibit a higher level of collinearity with the eudicots than with the monocots, suggesting that its gene order most closely resembles that of the eudicot ancestral genome because of a lack of genome duplication^60^. However, very low ratios were obtained between *A. trichopoda* and *B. rapa* for the flowering genes and the whole genome possibly because Chinese cabbage underwent a recent WGT event that resulted in rearrangement of its genes^55^. All of these phenomena may be related to genome duplication during the evolutionary history of the different species.

### Comparative analysis of *COL* genes in the whole Chinese cabbage genome

*CO* promotes the induction of flowering in *Arabidopsis* under long photoperiods and is one of the most important *COL* transcription factor families. Twenty-five *BraCOL* genes were identified in Chinese cabbage. All the *BraCOL* genes contained one to two B-box domains located in the N-terminus but had only one CCT domain, which comprised approximately 45 amino acids, was located in the C-terminus of the proteins, and was more conserved than the B-box domain. In addition, 34 *COL* genes identified in *Arabidopsis* and rice were selected for comparative analysis. The six most conserved motifs in the *BraCOL* genes were detected by MEME (Supplementary Fig. S3). Most of the *BraCOL* genes had motifs 2, 3, and 6, which correspond to highly conserved B-box domains. However, no motif 3 was detected in the group B proteins because these only contained one B-box domain. Motifs 1 and 4 were almost always found in all the *BraCOL* genes, and these constituted the CCT domain. In addition, the results from gene structure analyses were mostly consistent with those obtained from the analysis of gene motifs, and the members of the same branch usually shared a common intron or exon pattern (Supplementary Fig. S3).

To classify *BraCOL* genes, 25 *BraCOL* genes and 17 *AthCOL* genes were used to construct a phylogenetic tree (Supplementary Fig. S4). The *BraCOL* genes were clearly grouped into three groups according to their bootstrap values for phylogenetic relationship and classification in *Arabidopsis*^61^. Most of the *BraCOL* genes belonged to group C (11 genes), followed by groups A (eight genes) and B (six genes) (Supplementary Table S5). Genes with high sequence similarity generally have similar functions across different species. Thus, the construction of the phylogenetic tree of *BraCOL* genes based on *Arabidopsis* aids the determination of the functions of *BraCOL* genes. The phylogenetic analysis provides a solid foundation for future functional studies involving comparisons with *AthCOL* genes.

**Figure 2:**
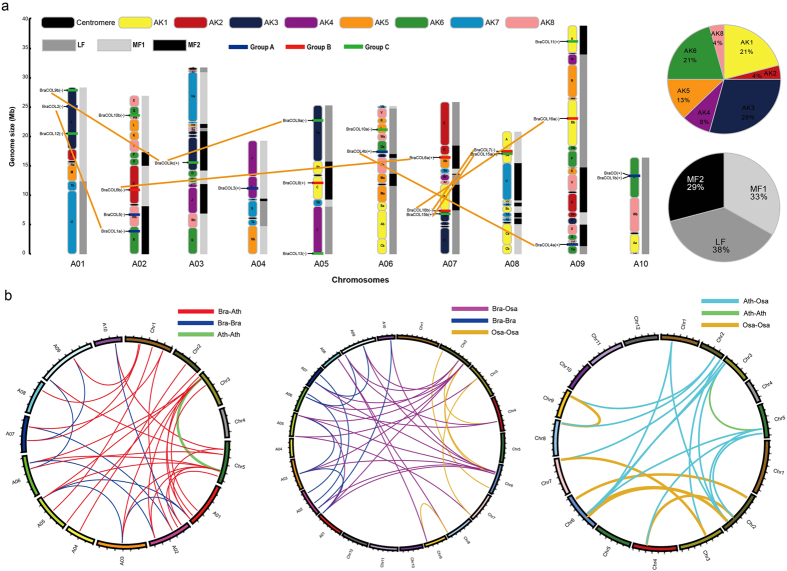
The features of the *COL* genes in *B. rapa*. (**a**) The distribution of *BraCOL* genes on ten chromosomes. The 8 ancestral blocks and three subgenomes, including the least fractionated (LF), medium fractionated (MF1), and most fractionated (MF2) subgenomes, were plotted as described by Cheng *et al.* (2013). AK represents the ancestral karyotype. The orange lines connect the duplicated *BraCOL* genes. (**b**) The following three maps were constructed based on the orthologous and paralogous pair positions: Chinese cabbage (A01–A10) and *Arabidopsis* chromosome (Chr1–Chr5) maps (left), Chinese cabbage (A01–A10) and rice chromosome (Chr1–Chr12) maps (middle), and *Arabidopsis* (Chr1–Chr5) and rice chromosome (Chr1–Chr12) maps (right). The red lines represent the orthologous pairs between Chinese cabbage and *Arabidopsis*, the purple lines represent the orthologous pairs between Chinese cabbage and rice, and the light blue lines represent the orthologous pairs between *Arabidopsis* and rice. The blue, green, and orange lines represent the paralogous pairs in Chinese cabbage, *Arabidopsis*, and rice, respectively.

Most *BraCOL* genes could be mapped onto ten chromosomes of Chinese cabbage with a non-random distribution (Fig. 2a). However, *BraCOL14* is located in Scaffold000467. Chromosome 2 contained more *BraCOL* genes (four genes) than the other chromosomes, whereas chromosomes 3 and 4 only contained one *BraCOL* gene. *Arabidopsis* has undergone two whole-genome duplications (WGD: α and β) and one whole-genome triplication event (WGT: γ). *B. rapa* shares this evolutionary history with *Arabidopsis* but has undergone an additional WGT event. Therefore, the *B. rapa* genome was further divided into three differentially fractionated subgenomes, namely the least fractionated (LF), medium fractionated (MF1), and most fractionated (MF2) subgenomes^55^. In this study, 24 *BraCOL* genes were mapped onto chromosomes fractionated into these three subgenomes: nine (38%) in LF, eight (33%) in MF1, and seven (29%) in MF2 (Fig. 2a). In addition, we reconstructed the *B. rapa* genome containing 24 conserved chromosomal blocks (labelled A–X) according to previous reports. The colour coding of these blocks was based on their positions in a proposed ancestral karyotype (AK1-8)^62^. Most of the *BraCOL* genes belonged to AK3 (29%), followed by AK1 and AK6 (21%), whereas no *BraCOL* gene was identified in AK7 (Fig. 2a). In Chinese cabbage, we identified 14 duplicated *BraCOL* genes, which were located in synteny regions (Supplementary Fig. S5). All of these duplicated genes were divided into six groups (Fig. 2a). Four of these groups each contained two duplicated genes, whereas two groups contained three duplicated genes. The groups contained only twoduplicated genes, which may be explained by the gene loss that accompanied genome triploidization in Chinese cabbage. Furthermore, the types and divergence time of the duplicated genes were estimated by calculating the numbers of synonymous substitutions (Ks). The Ks, Ka, and Ka/Ks values for seven Chinese cabbage and two *Arabidopsis* duplication pairs were calculated (Table 2). All the duplicated gene pairs belonged to genes that had undergone a WGD or segmental duplication, and all the duplicated *COL* gene pairs had a Ka/Ks ratio less than 1, indicating the purifying selection of these genes. The calculation of the divergence time of the duplicated *COL* genes revealed that it spanned 8.49 to 28.88 MYA (Table 2). The divergence time of *BraCOL9b* and *BraCOL9c* was 8.49 MYA, which indicates that their divergence occurred during the *Brassica* triplication events (5 ~ 9 MYA)^63^. However, the values obtained for the two duplication pairs in *Arabidopsis* and one duplication pair (*BraCOL2*- *BraCOL1a*) were greater than 20 MYA, which indicates that these duplications occurred during the α-tetraploidy event (20–40 MYA)^63^. The divergence time obtained for the other six duplication pairs ranged from 11.33 to 13.79 MYA, indicating that these duplications occurred during the divergence of Chinese cabbage and *Arabidopsis* (9.6–16.1 MYA)^55^. In addition, we also calculated the divergence times for the 14 duplication pairs between Chinese cabbage and *Arabidopsis* (Table 2) and found that it ranged from 10.37 to 30.04, indicating that these genes underwent differentiation before their triplication events^63^.

A comparative analysis was performed to identify orthologous and paralogous *COL* genes for the assessment of triplication in Chinese cabbage (Fig. 2b, Supplementary Table S6). Among all the *COL* genes, 27 orthologous gene pairs were identified between Chinese cabbage and *Arabidopsis*. Conversely, only 21 orthologous gene pairs were found between Chinese cabbage and rice, and 13 orthologous gene pairs were identified between *Arabidopsis* and rice. The number of orthologous genes between Chinese cabbage and *Arabidopsis* was greater than that between Chinese cabbage and rice, which is consistent with the close relationship between Chinese cabbage and *Arabidopsis*. This analysis showed that a more distant genetic relationship is associated with fewer orthologous genes between the two species. Our analysis may provide a new resource for comparing the relationships among different species. Additionally, nine and six paralogous *COL* gene pairs were identified in Chinese cabbage and rice, respectively, whereas only one paralogous *COL* gene pair was found in *Arabidopsis*. Among the nine COL gene pairs of Chinese cabbage, five pairs were also duplicated, which demonstrated that these five paralogous pairs were also located in the synteny region.

### The evolutionary pattern and origin of *COL* genes in plants

The COL gene family members are subdivided into three classes, called groups A to C. A previous report showed that these groups evolved before the divergence of gymnosperms and angiosperms and indicated that the COL genes in the Brassicaceae family evolved rapidly^40^. To investigate the evolution of the *COL* gene family in the plant kingdom, we selected 34 Angiospermae (27 eudicots, six monocots and one basal angiosperm), three Gymnospermae, one Pteridophyta, one Bryophyta and six Chlorophyta species for comparative analysis (Supplementary Table S7). A total of 538 *COL* genes were identified in all of these species using the Pfam program (Supplementary Table S8). The number of *COL* genes in most angiosperms was greater than that in the other species, potentially because most angiosperms have undergone genome duplication, which leads to gene expansion. Surprisingly, *Physcomitrella patens* contained 17 *COL* genes, which is a greater number than those found in the other lower plants. We constructed phylogenetic trees of the *COL* genes to analyse the evolutionary relationships of these species (Supplementary Fig. S6). The results showed that the *COL* genes of Chlorophyta were divided into two groups and separated from those of the other species. However, the *COL* genes of the Bryophyta, Pteridophyta and Gymnospermae species were mixed with those of the Angiospermae species and divided into three groups. In addition, all the *COL* genes of the Angiospermae species were divided into three groups but were separated based on the monocot and eudicot classification (Supplementary Fig. S7), which may be due to the sequence differentiation obtained after the divergence of monocots and eudicots.

In general, the group A genes have two zinc finger B-boxes, whereas the group B and C genes have one B-box, and the group C genes have an additional diverged zinc finger. However, Chlorophyta only contained two groups of *COL* genes (G1 and G2), which had one and two B-box domains (Supplementary Fig. S8). In Chlorophyta, a relatively lower plant, the *COL* genes were not completely separated based on their number of B-box domains. All the *COL* genes in the analysed Bryophyta, Pteridophyta, Gymnospermae and basal Angiospermae species can be divided into three groups defined using the above-mentioned classification (Supplementary Fig. S8). These results lead one to question whether the one B-box domain in group B originated from G1 or G2. To answer this question, we constructed a phylogenetic tree of these *COL* genes (Supplementary Fig. S9a). The results showed that G1 had a closer relationship with group B than G2. In addition, the six most conserved motifs were detected in these genes, and all group B and G1 genes contained motif 1. However, only a few G2 genes contained motif 1, whereas the other G2 genes contained motif 5 (Supplementary Fig. S9a,b). Furthermore, we also calculated the genetic distance among these three groups and found that the group B genes presented the shortest genetic distance, followed by G1 and G2. The genetic distance between the group B and G1 genes was shorter than that between the group B and G2 genes (Supplementary Fig. S9c). All of these results indicated that group B had a close relationship with G1 and may have originated from G1.

**Figure 3:**
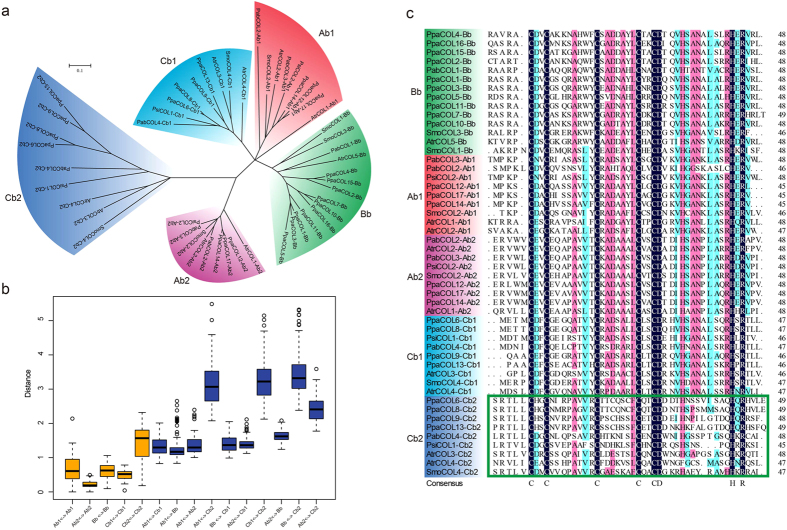
The analysis of the B-box domains in Bryophyta, Pteridophyta, Gymnospermae and basal Angiospermae. Ab1 and Ab2 represent the two B-box domains of group A, Cb1 and Cb2 represent the two B-box domains of group C, and Bb represents the one B-box domain of group B. (**a**) The phylogenetic tree of these five B-box sequences. (**b**) The genetic distance among the five B-box sequences. (**c**) The multiple sequence alignment of the five B-box domains. The abbreviations represent the species as follows: *Atr, Amborella trichopoda*; *Pta, Pinus taeda*; *Psi, Picea sitchensis*; *Pab, Picea abies*; *Smo, Selaginella moellendorffii*; *Ppa, Physcomitrella patens*.

To analyse the relationships of the B-box domains in Bryophyta, Pteridophyta, Gymnospermae and basal Angiospermae, we extracted the sequence of the B-box domains. The group A genes have two B-box domains, denoted Ab1 and Ab2, and the same result was obtained for the group C genes (Cb1 and Cb2). Group B contained only one B-box domain, named Bb. The phylogenetic tree showed that these B-box sequences were clearly divided into five classes (Fig. 3a). The results showed that the divergence in the Cb2 domain was greater than that found for the other four B-box domains. The genetic distance between Cb2 and the other four B-box domains was very large (Fig. 3b). This phenomenon could be verified by the alignment of multiple B-box sequences, and there was less conservation in Cb2 than in the other four B-box domains (Fig. 3c). We also constructed a phylogenetic tree for these four types of species using the sequences of their CCT domains, and the resulting tree was divided into three groups (Supplementary Fig. S10a). The MEME results showed that the CCT domain was more conserved than the B-box domain. Most CCT domains contained motif 1, and only three CCT domains of the group B *COL* genes exhibited a slight divergence (Supplementary Fig. S10a,b). The genetic distance analysis showed that group A was the most conserved among the three groups (Supplementary Fig. S10c). Although the CCT domain of the three groups was markedly conserved, we also identified several group-specific amino acids, including those in positions 25, 29, 31, and 34 in the multiple sequence alignment (Supplementary Fig. S10d). In addition, the phylogenetic tree also showed that the genes in groups A and B exhibited a close relationship (Supplementary Fig. S10e).

In angiosperms, we found that several *COL* genes were assigned to group A or group C according to their classification in *Arabidopsis* but had only one B-box domain, which is not consistent with the traditional definition. This phenomenon was only observed in some angiosperms. We then questioned why these genes that were classified as group A or C genes contain only one B-box domain; i.e., why they were assigned to group A or C and not to group B, which generally consists of the genes with only one B-box domain. To answer this question, we constructed a phylogenetic tree using the B-box domain sequences of several angiosperms, including *B. rapa, Arabidopsis* and rice (Supplementary Fig. S11a). The group A or C genes only contained one B-box domain, which we denoted Ab or Cb, respectively. The phylogenetic tree showed that all the Ab and Ab2 sequences clustered together and that all the Cb and Cb1 sequences were assigned to a group. To further analyse the relationship between these B-box domains, we calculated the genetic distances among them (Supplementary Fig. S11b). The results indicated that the genetic distance between Ab and Ab2 was shorter than that between Ab and Ab1 and that between Ab and Bb, and the genetic distance between Cb and Cb1 was shorter than that between Cb and Cb2 and that between Cb and Bb. This result was also consistent with the phylogenetic tree.

**Figure 4:**
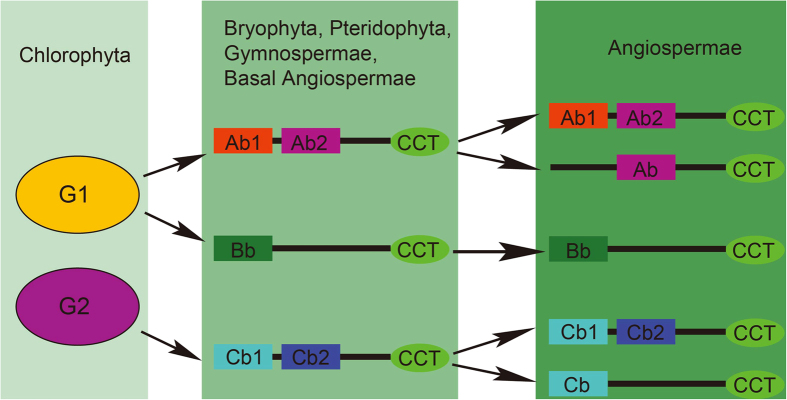
The evolutionary history pattern of *COL* genes in the plant kingdom. The tick and cross marks indicate that group B originated from G1. Ab and Cb represent the B-box domains of group A and group C genes that contained only one B-box domain.

Based on these results, we speculated that the Ab1 domain of several *COL* genes in group A was lost as a result of genome or gene duplication. However, the Cb2 domain of several *COL* genes in group C was lost during species evolution. This speculation is consistent with the theory of angiosperm genome duplication and loss. Therefore, we constructed the pattern underlying the evolutionary history of *COL* genes in the plant kingdom based on our findings (Fig. 4). Furthermore, we also calculated the percentage of B-box loss in group A or group C genes among these species (Table 3) and found that the percentages of B-box loss in groups A and C ranged from 0 to 17.65% (rice) and from 0 to 33.33% (*Panicum virgatum*), respectively. This phenomenon indicated that the percentage of B-box loss differed between the Angiosperm species due to their different evolutionary mechanisms.

### Comparative analysis of *CO* and *FT* genes between *Brassicaceae* and *Grass* species

*CO* is a central regulator of the photoperiod pathway because it triggers the production of the mobile florigen hormone *FT*, which induces flower differentiation^64^. It is an important member of the *COL* gene family, and 17, 16, 9 and 23 *COL* genes have been identified in Arabidopsis, rice, barley and cotton, respectively^17^,^52^. The flowering transition is regulated by *FT* and *TERMINAL FLOWER 1* (*TFL1*). *FT* promotes the transition to reproductive development and flowering, whereas *TFL1* represses this transition^53^. The common ancestor of the FT and TFL1 subfamilies functionally diverged from the MOTHER OF FT AND TFL1 (MFT) subfamily to activate and repress flowering, respectively^54^. The *Brassicaceae* species are typically eudicots, whereas grass is a typical monocot. Therefore, we surveyed the differences in *CO* and *FT* between *Brassicaceae* and *Grass* species and compared the protein, full gene and promoter sequences of five *Brassicaceae* species and three *Grass* species to uncover the factors affecting this regulatory mechanism.

**Figure 5:**
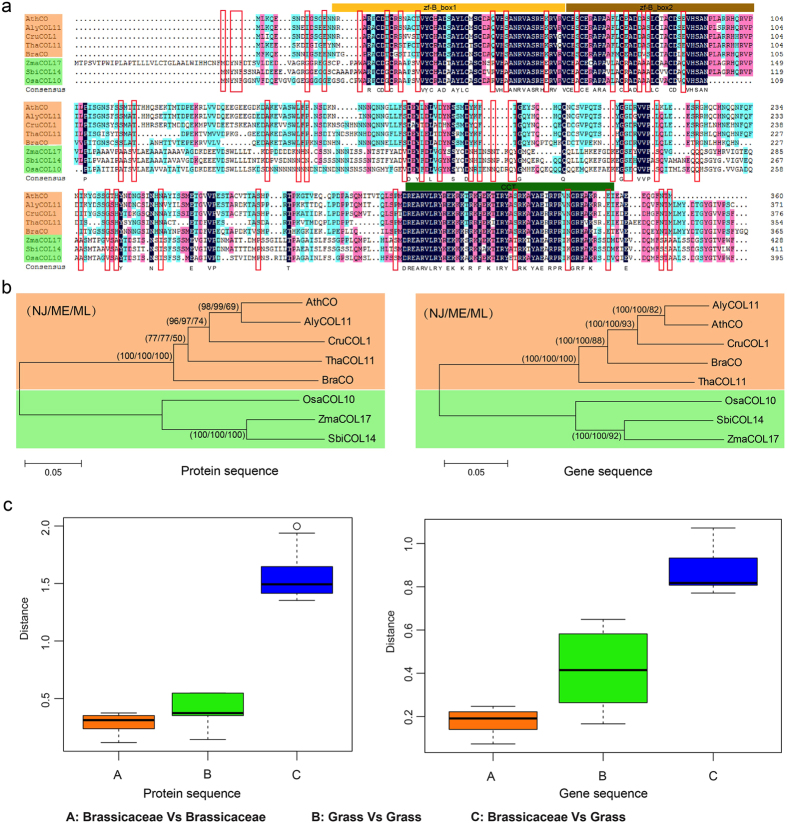
The comparative analysis of *CO* genes between *Brassicaceae* and *Grass* species. (**a**) The multiple sequence alignment of *CO* genes. The orange solid rectangle represents the five *Brassicaceae* species, and the green solid rectangle represents the three *Grass* species. The red hollow rectangle indicates the family-specific amino acids. (**b**) The phylogenetic trees were constructed using the *CO* protein and gene sequences. The numbers on the branches of the phylogenetic tree represent the bootstrap supports provided by the NJ, ME and ML methods. (**c**) The genetic distance in *Brassicaceae* and *Grass* was estimated using the *CO* protein and gene sequences.

**Figure 6:**
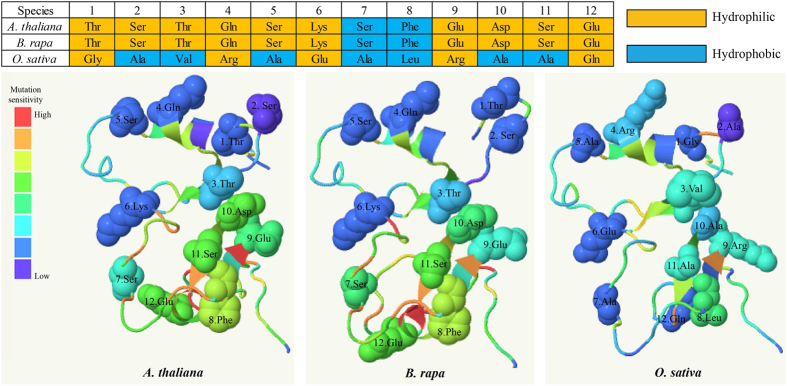
The protein structure of CO in *Arabidopsis*, Chinese cabbage and rice. The nine family-specific amino acids located in the two B-box domains are marked on the protein structure. The orange solid rectangle represents the hydrophilic amino acids, and the blue solid rectangle indicates the hydrophobic amino acids.

The multiple sequences of *CO* revealed 48 family-specific amino acids between *Brassicaceae* and *Grass* (Fig. 5a). Among these, 13 sites were located in the two B-box domains, and three sites were located in the CCT domain. The phylogenetic trees constructed based on the protein and gene sequences exhibited similar topology (Fig. 5b). The sequences were divided into two groups, one corresponded to the sequences found in *Brassicaceae* species, and the other comprised the sequences in *Grass* species. This phenomenon indicated that *CO* was highly conserved in *Brassicaceae* and in *Grass*. The genetic distance calculated between the two sequence types confirmed this finding (Fig. 5c). The genetic distance between *Brassicaceae* and *Grass* was greater than that obtained between *Brassicaceae* species and that obtained between *Grass* species. Furthermore, we predicted a 3D model of the CO proteins in *B. rapa, Arabidopsis* and rice. The confidence of the protein structure prediction reached 97% in these three species. Twelve family-specific amino acids were located in the two B-box domains on the predicted protein structure (Fig. 6). Overall, the structures found for CO proteins in *B. rapa* and *Arabidopsis* were similar, but these were quite different from that found in rice CO proteins. The mutation sensitivity of sites 8, 10, 11 and 12 was higher than that obtained for other sites in *Arabidopsis* and *B. rapa*. However, only site 8 exhibited a higher mutation sensitivity than the other sites in rice. In addition, sites 2, 3, 5, 10 and 11 were associated with hydrophilic amino acids in *B. rapa* and *Arabidopsis* and with hydrophobic amino acids in rice. Therefore, these amino acids may be more likely to affect the function of the CO proteins.

The *FT* genes in five Brassicaceae and three Grass species were investigated in this study (Supplementary Table S9). The genes with the most sequence similarity with *Arabidopsis FT* gene were defined as the FT genes in each Brassicaceae and Grass species. These genes were representative members of each species FT family. For example, *LOC_Os06g06320* gene corresponded to *Hd3a* in rice, and was chosen for the FT comparative analysis. The multiple sequences of *FT* revealed 27 family-specific amino acids between *Brassicaceae* and *Grass* (Supplementary Fig. S12a). The phylogenetic trees constructed based on the protein and gene sequences had similar topology (Supplementary Fig. S12b). We also predicted the 3D model of FT proteins in *B. rapa, Arabidopsis* and rice. The confidence of the protein structure prediction reached 100% in these three species. Twenty-two family-specific amino acids were located in the predicted protein structures (Supplementary Fig. S13). The mutation sensitivity of sites 17 and 19 was higher than that obtained for the other 17 sites in *Arabidopsis*. However, with the exception of sites 19 and 22, site 12 also exhibited a high mutation sensitivity in *B. rapa*. In rice, the mutation sensitivity of sites 12, 15 and 22 was higher than that of the other 19 sites. Furthermore, sites 1, 4, 15 and 22 were associated with hydrophobic amino acids in *B. rapa* and *Arabidopsis* and with hydrophilic amino acids in rice. Sites 7 and 16 were associated with hydrophilic amino acids in *B. rapa* and *Arabidopsis* but with hydrophobic amino acids in rice. Therefore, these amino acids may be more likely to affect FT protein function.

### Expression analysis of photoperiod pathway- and circadian clock-related genes

The flowering of *Arabidopsis* is promoted by long days and is repressed by short days. Chinese cabbage is a member of the *Brassica* genus and is one of most important vegetables cultivated worldwide. It has experienced thousands of years of cultivation and artificial selection. The genome has undergone triplication events since its divergence from *Arabidopsis*; however, high degrees of sequence similarity and genome structure conservation remain between the two species. The flowering genes in *Arabidopsis* have been well studied, making *Arabidopsis* a viable reference species for comparative genomic studies. Variations in the number of flowering genes due to genome triplication may contribute to the broad range of flowering time plasticities observed in *Brassica* species.

**Figure 7:**
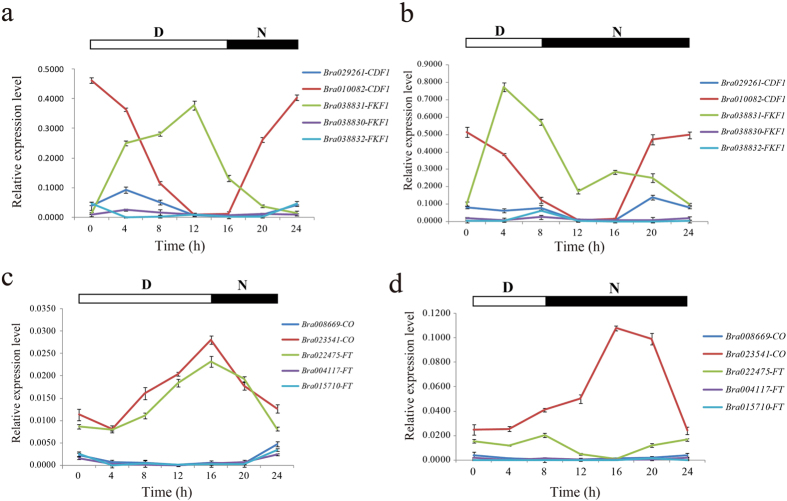
The analysis of the expression of photoperiod pathway and circadian clock-related genes by RT-qPCR. The bars correspond to the means ± S.D. from three independent experiments. D and N represent day and night, respectively. The RNA expression level relative to the *actin* gene was calculated using the 2^−ΔΔCT^ method and was thenlog2-transformed. (**a**) The relative expression of *CDF1* and *FKF1* genes in Chinese cabbage under LD conditions over a one-day period. (**b**) The relative expression of *CDF1* and *FKF1* genes in Chinese cabbage under SD conditions over a one-day period. (**c**) The relative expression of *CO* and *FT* genes in Chinese cabbage under LD conditions over a one-day period. (**d**) The relative expression of *CO* and *FT* genes in Chinese cabbage under SD conditions over a one-day period.

To investigate the expression pattern of the regulatory network of photoperiod pathway- and circadian clock-related genes in *B. rapa*, we measured the expression of these genes under different photoperiod treatments by RT-qPCR^65^. We compared the expression levels of these genes under LD and SD conditions over a four-week period (Supplementary Table S10). The results showed that most genes were up-regulated at night during the first week and down-regulated during the third week (Supplementary Fig. S14). The expression levels of these genes during the day and night differed, which indicated that they were very sensitive to light or were controlled by the circadian clock. In addition, we also surveyed the expression levels of these genes at different times over a period of one day under LD and SD conditions. Compared with *Arabidopsis*, several genes were duplicated in *B. rapa*, but only one duplicated gene in each of the duplicated pairs exhibited high expression. For example, there were three FKF1 genes in *B. rapa*, but only the *Bra038831-FKF1* gene was highly expressed, whereas the other two *FKF1* genes were less strongly expressed (Fig. 7b). A similar phenomenon was found for other duplicated gene pairs, such as *CDF1, CO, FT, LHY, TOC1* and *COP1* (Fig. 7a–d, Supplementary Fig. S15). The expression of the two *PRR7* genes was lower than the expression of the two *PRR9* genes in *B. rapa*. The *PRR7* genes presented similar expression trends under LD conditions, whereas the opposite expression trend was observed under SD conditions (Supplementary Fig. S15a,b). Under LD and SD conditions, the expression trend for *TOC1* contrasted with that obtained for *LHY* genes (Supplementary Fig. S15c,d). *Bra005541-COP1* and the two *CRY1* genes exhibited similar expression trends, whereas the *Bra021818-COP1* gene was hardly expressed (Supplementary Fig. S15e,f). The expression of the *PHYB* gene was higher than the expression levels of the *PHYA* genes under LD and SD conditions (Supplementary Fig. S15g,h). The expression trend detected for *Bra038831-FKF1* and *Bra010082-CDF1* was the opposite of that obtained under LD conditions, whereas their expression levels were similar between 4 h to 16 h under SD conditions (Fig. 7b,c). The expression trends obtained for *Bra023541-CO* and *Bra022475-FT* were the same under LD conditions, whereas their expression trends were the opposite between 8 h and 24 h under SD conditions (Fig. 7d,e). Interestingly, all three duplicated *SOC1* genes were expressed, whereas their expression trends were reversed under LD and SD conditions (Supplementary Fig. S15i,j).

## Conclusion

In this study, we analysed the evolutionary pattern, gene duplication, and expression level of the key genes involved in the photoperiod pathway. We compared the flowering genes in monocots and eudicots, and this analysis included the identification of the family-specific genes as well as the orthologous and collinear genes. The *BraCOL* genes in the Chinese cabbage genome were identified and subjected to a systematic analysis. Furthermore, the evolution and origin of the *COL* genes in the plant kingdom were analysed, and the evolutionary pattern of the *COL* genes was determined. In addition, we compared the *CO* and *FT* genes between *Brassicaceae* and *Grass* species. Moreover, the expression of photoperiod pathway- and the circadian clock-related genes in *B. rapa* under different photoperiod treatments were analysed by RT-qPCR. Because the photoperiod pathway- and circadian clock-related genes in *B. rapa* have not been previously studied, the expression analysis presented in this article provides a solid foundation for future functional studies. In conclusion, this study provides useful resources for future studies on the structure and function of flowering genes and for identifying and characterizing flowering genes in other species. In addition, this study may also facilitate our understanding of the effect of polyploidy during the evolution of flowering genes.

## Methods

### Retrieval of Sequences

The *Arabidopsis* sequences were retrieved from TAIR (http://www.arabidopsis.org/), and the sequences of rice were retrieved from RGAP (http://rice.plantbiology.msu.edu/)^66^. The *B. rapa* sequences were downloaded from BRAD (http://brassicadb.org/brad/)^67^. The genome sequences of *A. trichopoda* were downloaded from the Amborella Genome Database (http://amborella.huck.psu.edu/)^60^. In addition, the protein sequences of *Gymnospermae* species, including *P. taeda, P. sitchensis* and *P. abies*, were downloaded from the Forest Tree Genome database (http://dendrome.ucdavis.edu/treegenes/)^68^, and the sequences of the other 38 species used in this study were downloaded from JGI (http://www.phytozome.net/)^69^. All of these species are representative species of the different branches in the plant phylogenetic tree. The homologous flowering genes in other species were identified through comparison with *Arabidopsis*. First, BLASTP searches were performed against the rice protein sequences using an E-value threshold of 1 × 10^−10^. The top-ranked rice hit was used for BLASTP searches of the *Arabidopsis* proteins to confirm homologies. Starting with both *Arabidopsis* and rice homologues, BLASTP searches were performed against the proteins of other species (e-value <1 × 10^−10^, identity >40%). The putative orthologous flowering genes were confirmed by reciprocal BLASTP searches of the *Arabidopsis* and rice protein sequence datasets (Fig. 1a, Supplementary Table S1). Information regarding genome duplication or triplication was obtained from the Plant Genome Duplication Database (PGDD)^70^. A flow chart of this study was drawn using Microsoft Visio (Supplementary Fig. S16).

### Identification of gene synteny and duplicated *CO* genes

BLAST and the Multiple Collinearity Scan toolkit (MCScanX) were used for gene synteny analysis according to previous reports^59^,^71^. The duplicated *CO* genes were identified using MCScanX. First, the whole-genome protein sequences from Chinese cabbage, *Arabidopsis*, and rice were searched against themselves using BLASTP with an E-value cut-off of 1 × 10^−10^ and identity >75%^72^. MCScanX was then used for detecting synteny regions according to a previous report^59^. We then identified the duplicated *CO* genes from these duplicated regions. The Venn diagrams were drawn using the R program.

### Phylogenetic analysis of COL and FT family genes

Phylogenetic analyses were conducted using MEGA v5.0^73^. Neighbour-joining (NJ), maximum-likelihood (ML) and minimum-evolution (ME) trees were constructed with a bootstrap value of 1,000 replications to assess the reliability of the resulting trees. For analysis of the *COL* genes, the B-box and CCT domains of the protein sequences were used to construct phylogenetic trees for the different species. In addition, the full amino acid and DNA sequences of the *CO* and *FT* genes were used to construct a tree for the analysis of the relationship between *Brassicaceae* and *Grass* species. The genetic distance used in this study was also calculated with MEGA v5.0.

### Characterization of genes of the *COL* and *FT* families

The Pfam database was used to identify the genes belonging to the *COL* and *FT* families^74^. The Pfam accession number PF06203.9 was used for identifying the CCT domain, and. PF00643.19 was used for identification of the zf-B_box domain. The proteins containing these two conserved domains were defined as *COL* genes. PF01161.15 was used for identifying genes of the *FT* family. The genes identified by Pfam were further verified using SMART^75^. Conserved motifs were identified using MEME^76^, and the gene structures were obtained by GSDS (http://gsds.cbi.pku.edu.cn/)^77^. The genes with the most sequence similarity with *Arabidopsis CO* or *FT* genes were defined as the *CO* or *FT* genes in each *Brassicaceae* and *Grass* species, respectively. The full amino-acid and DNA sequences of the *CO* and *FT* genes were used to identify the family-specific sites in *Brassicaceae* and *Grass* species. The sequences were aligned using ClustalX 2.0^78^. The *CO* and FT protein structures were predicted using the Phyre2 program^79^.

### **Identification of orthologous and paralogous** ***COL*** **genes**

The orthologous and paralogous *COL* genes in Chinese cabbage, *Arabidopsis*, and rice were identified using OrthoMCl^56^. The relationships between orthologous and paralogous genes among the three species were plotted using Circos^80^. The position of the *COL* gene of Chinese cabbage was marked on a chromosome using an in-house-developed Perl script.

### Calculation of the Ka/Ks and dating of the duplication events

To estimate the divergence of the duplicated *COL* genes, the sequences of the duplicated pairs of *COL* genes were aligned using ClustalW2. We calculated the synonymous rate (Ks), non-synonymous rate (Ka), and evolutionary constraint (Ka/Ks) between the duplicated pairs of *COL* genes based on the alignments of their coding sequences obtained using the method developed by Nei and Gojobori, which is incorporated in the KaKs_calculator^81^. The divergence time was calculated using the formula T = Ks/2R, where Ks refers to the synonymous substitutions per site, and R is the rate of divergence of nuclear genes from plants. R was considered to equal 1.5 × 10^−8^ synonymous substitutions per site per year for dicotyledonous plants^82^.

### Plant materials, growth conditions, and photoperiod treatments

Seeds of the Chinese cabbage cultivar Chiifu-401–42 were grown in pots containing a soil:vermiculite mixture (3:1) in a controlled-environment growth chamber programmed for 16/8 h at 24/18 °C for day/night and a relative humidity of 60–65%. Seedlings at the five-leaf stage were transferred to growth chambers and exposed to different photoperiods. The photoperiod treatments were defined as short-day (8 hours of light/16 hours of dark) and long-day (16 hours of light/8 hours of dark), and the other conditions were not changed. After treatment for one week, we collected leaf samples at 6/20 h under the SD conditions, and at 12/22 h under the LD conditions using 0 h as the dawn. Samples were collected once a week for four weeks, when flowering occurred. In addition, we also obtained samples every 4 hours to survey the pattern of gene expression over a one-day period after two weeks of treatment. All the leaf samples were frozen in liquid nitrogen and stored at −70 °C.

### RNA isolation and RT-qPCR analyses

The RNA from the leaves was isolated using an RNA kit (Tiangen, Beijing, China) according to the manufacturer’s instructions. Using the PrimeScript™ RT Reagent Kit (TaKaRa, Kyoto, Japan), 1 μg of RNA was then used to synthesize cDNA for RT-qPCR, which was performed in a 20 μl reaction volume. The cDNA reaction mixture was diluted 1:10 with EASY Dilution for RT-qPCR, and 2 μl of this mixture was used as the template in the 20 μl PCR reactions. The *actin* gene (*Bra028615*) of Chinese cabbage was used as an internal control to normalize the expression levels of the target genes. Specific primers were designed according to the corresponding gene sequences using Primer 5.0 (Supplementary Table S11). The RT-qPCR assays were performed using three biological and technical replicates. Each reaction was performed in 20 μl reaction mixtures, including SYBR Premix Ex Taq (2×) (TaKaRa, Kyoto, Japan), gene-specific primers, and the diluted cDNA sample as the template. The RT-qPCR assay was performed according to a previous report^83^. The gene expression level relative to that of the *actin* gene was calculated as 2^−ΔΔCT^ according to a previous analysis^84^. The gene expression patterns of each tissue were analysed using Cluster3.0, and the expression values were log2-transformed. Heat maps were then constructed using Tree View (http://jtreeview.sourceforge.net/) for visualization of the hierarchical clustering results.

## Additional Information

**How to cite this article**: Song, X. *et al.* Comprehensive analysis of the flowering genes in Chinese cabbage and examination of evolutionary pattern of CO-like genes in plant kingdom. *Sci. Rep.* **5**, 14631; doi: 10.1038/srep14631 (2015).

## Supplementary Material

Supplementary Figures S1-S16

Supplementary Tables S1-S11

## Figures and Tables

**Table 1:**
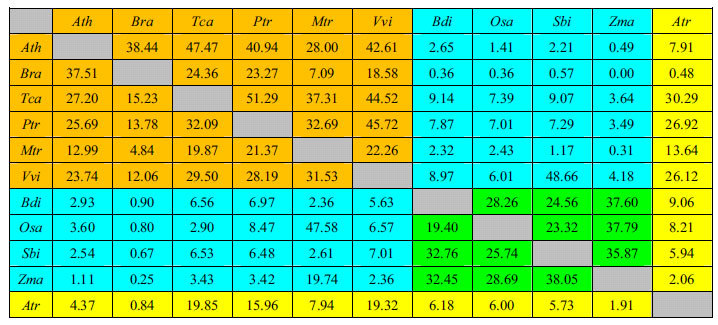
The numbers located below the diagonal represent the percentage of collinear ortholog pairs (collinear ortholog pairs number/number of all ortholog pairs) in the genomes of 11 angiosperms. The numbers located above the diagonal represent the percentage of collinear ortholog pairs in the flowering genes of 11 angiosperms.

Note: The abbreviations represent the species as follows: *Ath, Arabidopsis thaliana; Bra, Brassica rapa; Tca, Theobroma cacao; Ptr, Populus trichocarpa; Mtr, Medicago truncatula; Vvi, Vitis vinifera; Bdi, Brachypodium distachyon; Osa, Oryza sativa; Sbi, Sorghum bicolor; Zma, Zea mays; Atr, Amborella trichopoda*. The orange colour represents the percentage of collinear ortholog pairs within eudicot species, the green colour represents the percentage of collinear ortholog pairs within monocot species, the blue colour represents the percentage of collinear ortholog pairs between eudicot and monocot species, and the yellow colour represents the percentage of collinear ortholog pairs between Amborella trichopoda and other angiosperm species.

**Table 2:** Calculation of Ka/Ks and the divergence time of the duplicated *COL* gene pairs in Chinese cabbage and *Arabidopsis*.

Duplicated gene pairs	Ka	Ks	Ka/Ks	*P-Value*(Fisher)	Duplication type	Purify selection	Time (MYA)
*BraCOL2-BraCOL1a*	0.24	0.63	0.37	1.01E–09	WGD or segmental	Yes	21.14
*BraCOL9b-BraCOL9c*	0.05	0.25	0.18	6.68E–14	WGD or segmental	Yes	8.49
*BraCOL6b-BraCOL6a*	0.12	0.40	0.30	1.39E–13	WGD or segmental	Yes	13.23
*BraCOL9c-BraCOL9a*	0.04	0.41	0.10	1.18E–28	WGD or segmental	Yes	13.79
*BraCOL4b-BraCOL4a*	0.05	0.34	0.14	1.26E–21	WGD or segmental	Yes	11.39
*BraCOL16b-BraCOL7*	0.20	0.38	0.54	8.07E–05	WGD or segmental	Yes	12.63
*BraCOL16b-BraCOL16a*	0.08	0.36	0.22	1.81E–17	WGD or segmental	Yes	11.97
*BraCOL15a-BraCOL15b*	0.12	0.34	0.35	1.34E–23	WGD or segmental	Yes	11.33
*AthCOL2-AthCO*	0.17	0.61	0.29	1.23E–15	WGD or segmental	Yes	20.33
*AthCOL9-AthCOL10*	0.18	0.87	0.21	3.35E–27	WGD or segmental	Yes	28.88
*AthCOL2-BraCOL2*	0.11	0.31	0.34	2.67E–08	WGD or segmental	Yes	10.37
*AthCOL2-BraCOL1b*	0.25	0.70	0.35	5.92E–12	WGD or segmental	Yes	23.4
*AthCOL11-BraCOL12*	0.28	0.90	0.31	1.38E–15	WGD or segmental	Yes	30.04
*AthCO-BraCOL2*	0.21	0.69	0.30	4.81E–14	WGD or segmental	Yes	23.17
*AthCOL10-BraCOL10b*	0.10	0.52	0.19	1.11E–22	WGD or segmental	Yes	17.29
*AthCOL10-BraCOL10a*	0.12	0.61	0.20	1.24E–23	WGD or segmental	Yes	20.38
*AthCOL4-BraCOL4a*	0.04	0.79	0.05	3.84E–56	WGD or segmental	Yes	26.21
*AthCOL6-BraCOL6b*	0.12	0.40	0.30	5.03E–13	WGD or segmental	Yes	13.46
*AthCOL8-BraCOL8*	0.15	0.35	0.43	7.58E–06	WGD or segmental	Yes	11.52
*AthCOL6-BraCOL6a*	0.10	0.37	0.27	2.03E–14	WGD or segmental	Yes	12.38
*AthCOL16-BraCOL7*	0.23	0.66	0.35	7.07E–14	WGD or segmental	Yes	21.9
*AthCOL15-BraCOL15a*	0.16	0.73	0.23	5.14E–26	WGD or segmental	Yes	24.24
*AthCOL16-BraCOL16a*	0.08	0.46	0.18	4.96E–25	WGD or segmental	Yes	15.3
*AthCOL9-BraCOL9b*	0.07	0.39	0.18	9.26E–20	WGD or segmental	Yes	12.85

**Table 3:** The statistics of the B-box domain lost in group A and group C *COL* genes of angiosperm species.

	Group A		Group B	Group C		Total	Percentage*	Percentage*
*Species*	Two B-box	One B-box	One B-box	Two B-box	One B-box	Gene Number	Group A-lost	Group C-lost
*Aco*	2	0	1	5	0	8	0.00	0.00
*Aly*	6	0	4	5	3	18	0.00	16.67
*Ath*	6	0	4	7	0	17	0.00	0.00
*Bdi*	5	2	3	2	3	15	13.33	20.00
*Bra*	7	1	6	8	3	25	4.00	12.00
*Ccl*	3	1	2	8	0	14	7.14	0.00
*Cpa*	3	0	2	5	0	10	0.00	0.00
*Cru*	6	0	4	5	1	16	0.00	6.25
*Csa*	4	1	3	5	0	13	7.69	0.00
*Csi*	3	1	2	12	0	18	5.56	0.00
*Egr*	3	0	2	5	0	10	0.00	0.00
*Fve*	3	0	2	2	1	8	0.00	12.50
*Gma*	8	0	6	12	0	26	0.00	0.00
*Gra*	6	2	3	12	0	23	8.70	0.00
*Lus*	2	2	2	8	1	15	13.33	6.67
*Mdo*	6	0	5	6	0	17	0.00	0.00
*Mes*	7	0	3	7	1	18	0.00	5.56
*Mgu*	3	1	3	4	1	12	8.33	8.33
*Mtr*	4	0	1	4	1	10	0.00	10.00
*Osa*	4	3	3	2	5	17	17.65	29.41
*Ppe*	3	0	2	5	0	10	0.00	0.00
*Ptr*	6	0	3	7	3	19	0.00	15.79
*Pvi*	6	3	2	1	6	18	16.67	33.33
*Pvu*	4	0	4	6	0	14	0.00	0.00
*Rco*	3	0	2	4	1	10	0.00	10.00
*Sbi*	5	1	3	3	4	16	6.25	25.00
*Sit*	4	1	5	4	1	15	6.67	6.67
*Sly*	5	0	3	5	0	13	0.00	0.00
*Stu*	5	0	2	9	0	16	0.00	0.00
*Tca*	4	0	2	6	0	12	0.00	0.00
*Tha*	4	0	4	7	1	16	0.00	6.25
*Vvi*	2	0	1	3	0	6	0.00	0.00
*Zma*	5	2	5	4	1	17	11.76	5.88

Note: The abbreviations represent the species as follows: *Aco, Aquilegia coerulea*; *Aly, Arabidopsis lyrata*; *Ath, Arabidopsis thaliana*; *Bdi, Brachypodium distachyon*; *Bra, Brassica rapa*; *Ccl, Citrus clementina*; *Cpa, Carica papaya*; *Cru, Capsella rubella*; *Csa, Cucumis sativus*; *Csi, Citrus sinensis*; *Egr, Eucalyptus grandis*; *Fve, Fragaria vesca*; *Gma, Glycine max*; *Gra, Gossypium raimondii*; *Lus, Linum usitatissimum*; *Mdo, Malus domestica*; *Mes, Manihot esculenta*; *Mgu, Mimulus guttatus*; *Mtr, Medicago truncatula*; *Osa, Oryza sativa*; *Ppe, Prunus persica*; *Ptr, Populus trichocarpa*; *Pvi, Panicum virgatum*; *Pvu, Phaseolus vulgaris*; *Rco, Ricinus communis*; *Sbi, Sorghum bicolor*; *Sit, Setaria italica*; *Sly, Solanum lycopersicum*; *Stu, Solanum tuberosum*; *Tca, Theobroma cacao*; *Tha, Thellungiella halophila*; *Vvi, Vitis vinifera*; *Zma, Zea mays*.

^*^The percentage of B-box loss in Group A or Group C = Number of genes that contained only one B-box domain in Group A or Group C/All the *COL* genes in the species.
