# Supplementary material for: Comprehensive analysis of the flowering genes in Chinese cabbage and examination of evolutionary pattern of CO-like genes in plant kingdom
Source: Sci Rep. 2015 Sep 29;5:14631. doi: 10.1038/srep14631 (PMC4586889; doi:10.1038/srep14631)
Supplement: Supplementary Figures S1-S16 [file srep14631-s1.pdf]

Comprehensive analysis of the flowering genes in Chinese cabbage and examination of evolutionary pattern of CO-like genes in plant kingdom

Xiaoming Song[1, 2 †], Weike Duan[1, †], Zhinan Huang[1], Gaofeng Liu[1], Peng Wu[1], Tongkun Liu[1], Ying Li[1] & Xilin Hou[1, *]

[1]State Key Laboratory of Crop Genetics and Germplasm Enhancement/Key Laboratory of Biology and Germplasm Enhancement of Horticultural Crops in East China, Ministry of Agriculture, Nanjing Agricultural University, Nanjing 210095, China.

[2]Center of Genomics and Computational Biology, College of Life Sciences, North China University of Science and Technology, Tangshan, Hebei 063000, China.

[†]These authors contributed equally to this work.

*****Corresponding author: Email: hxl@njau.edu.cn

Supplementary Figures S1-S16

Genes	*A. thaliana*	*B. rapa*
AGL24	1	2
AP1	1	7
AP2	1	2
CDF1	1	2
CO	1	2
COP1	1	2
CRY1	1	2
CRY2	1	1
FCA	1	1
FKF1	1	3
FLC	1	4
FLD	1	1
FLK	1	1
FLM	1	0
FPA	1	1
FRI	1	1
FT	1	4
FVE	1	5
FY	1	2
GA1	1	2
GAI	1	2
GI	1	1
LD	1	1
LFY	1	2
PHYA	1	2
PHYB	1	1
RGA	1	2
SMZ	1	1
SNZ	1	0
SOC1	1	5
SPL	1	1
SPY	1	2
SVP	1	2
TOE1	1	2
TOE2	1	2
TOE3	1	1
VIN3	1	2
VRN1	1	3
VRN2	1	1

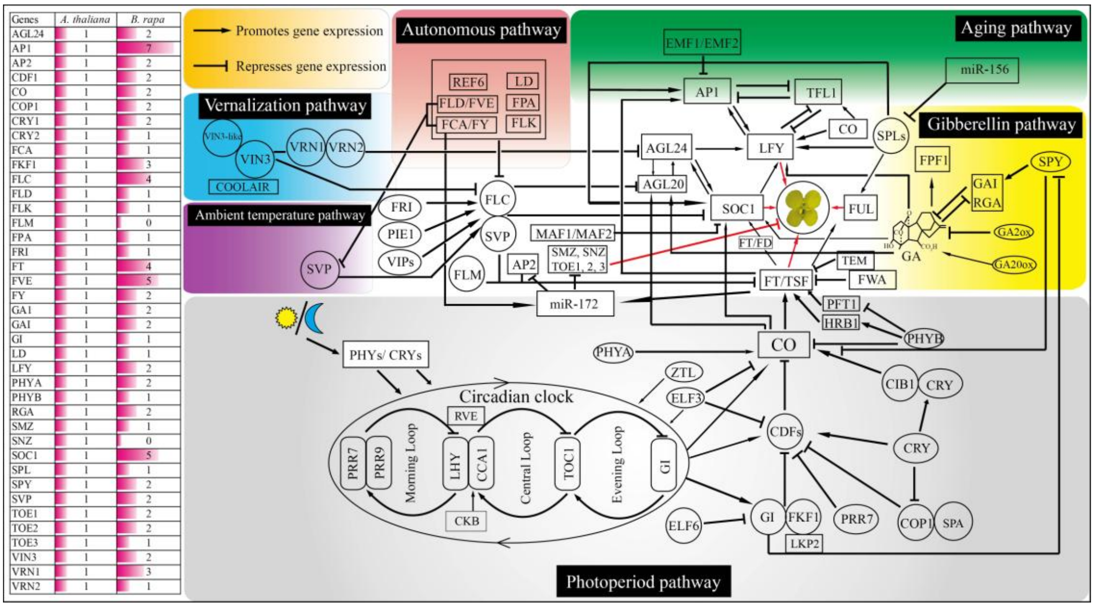

Figure S1. Major flowering pathways of *Arabidopsis*, including vernalization, photoperiod, gibberellin, autonomous, ambient temperature and aging pathways. The left of the figure shows the major flowering genes of *Arabidopsis* corresponding to the flowering genes in *B. rapa*.

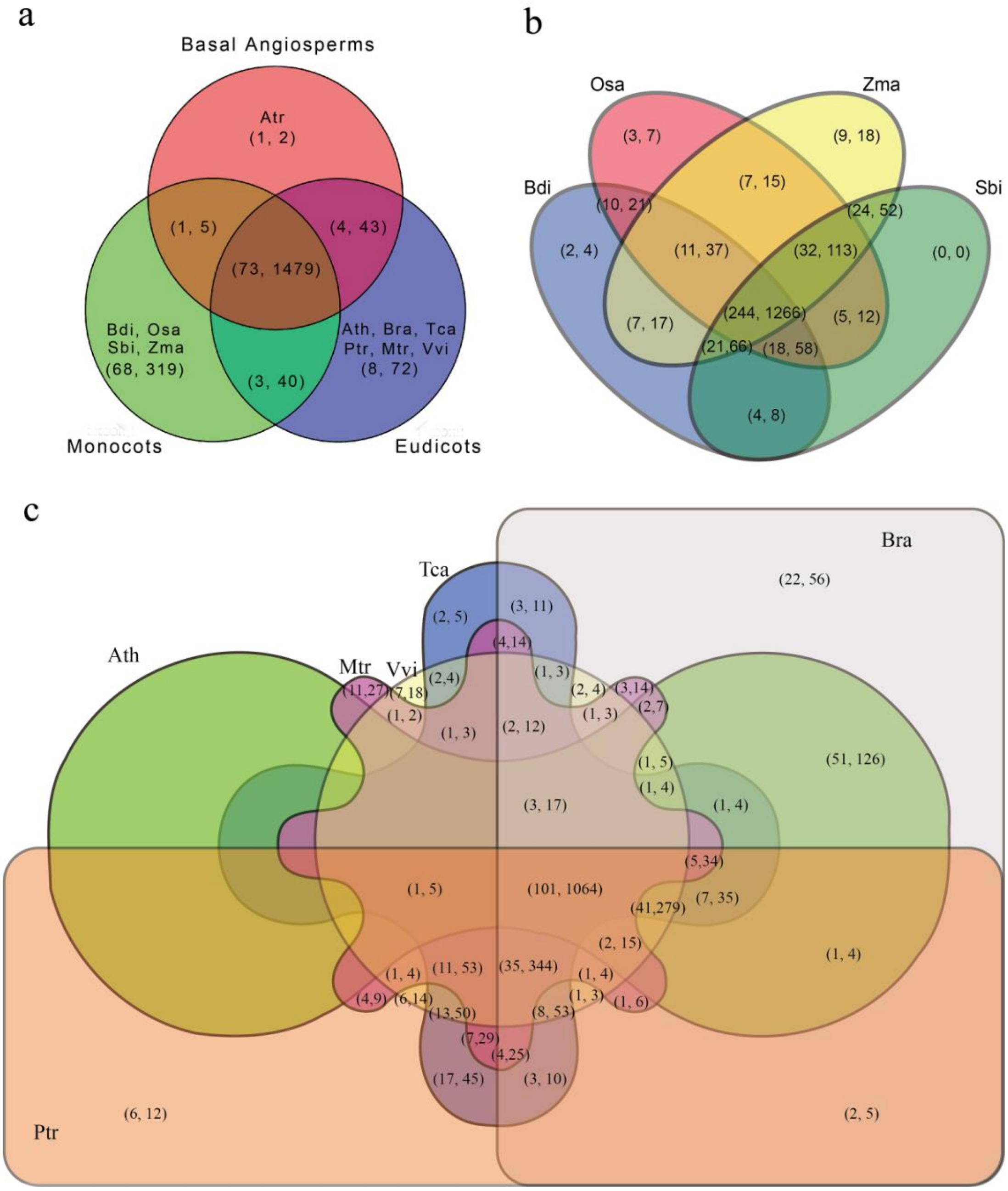

Figure S2. The Venn diagram shows the number of common and specific gene families and genes among the groups. The first number in the brackets represents the number of gene families, and the second number represents the number of genes. (a) Eudicots, monocots and a basal Angiosperm species. (b) Four monocot species. (c) Six eudicot species.

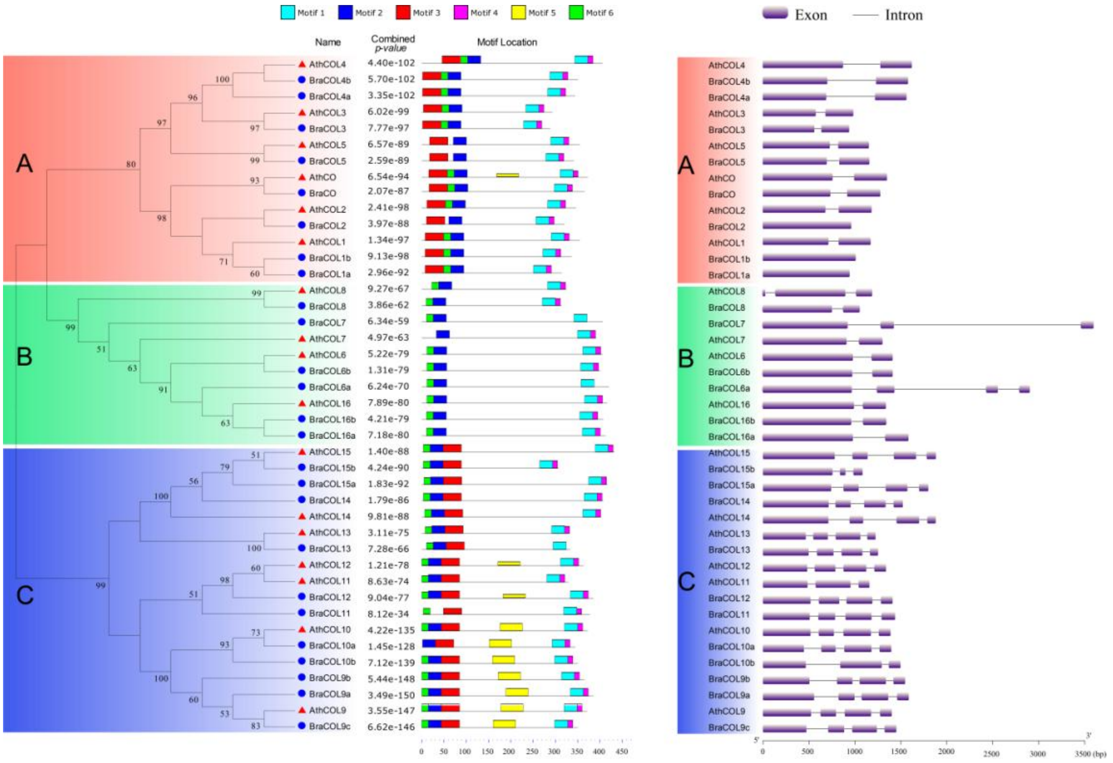

Figure S3. Schematic diagram of amino acid motifs and gene structures of *COL* genes in *Arabidopsis* and *B. rapa*.

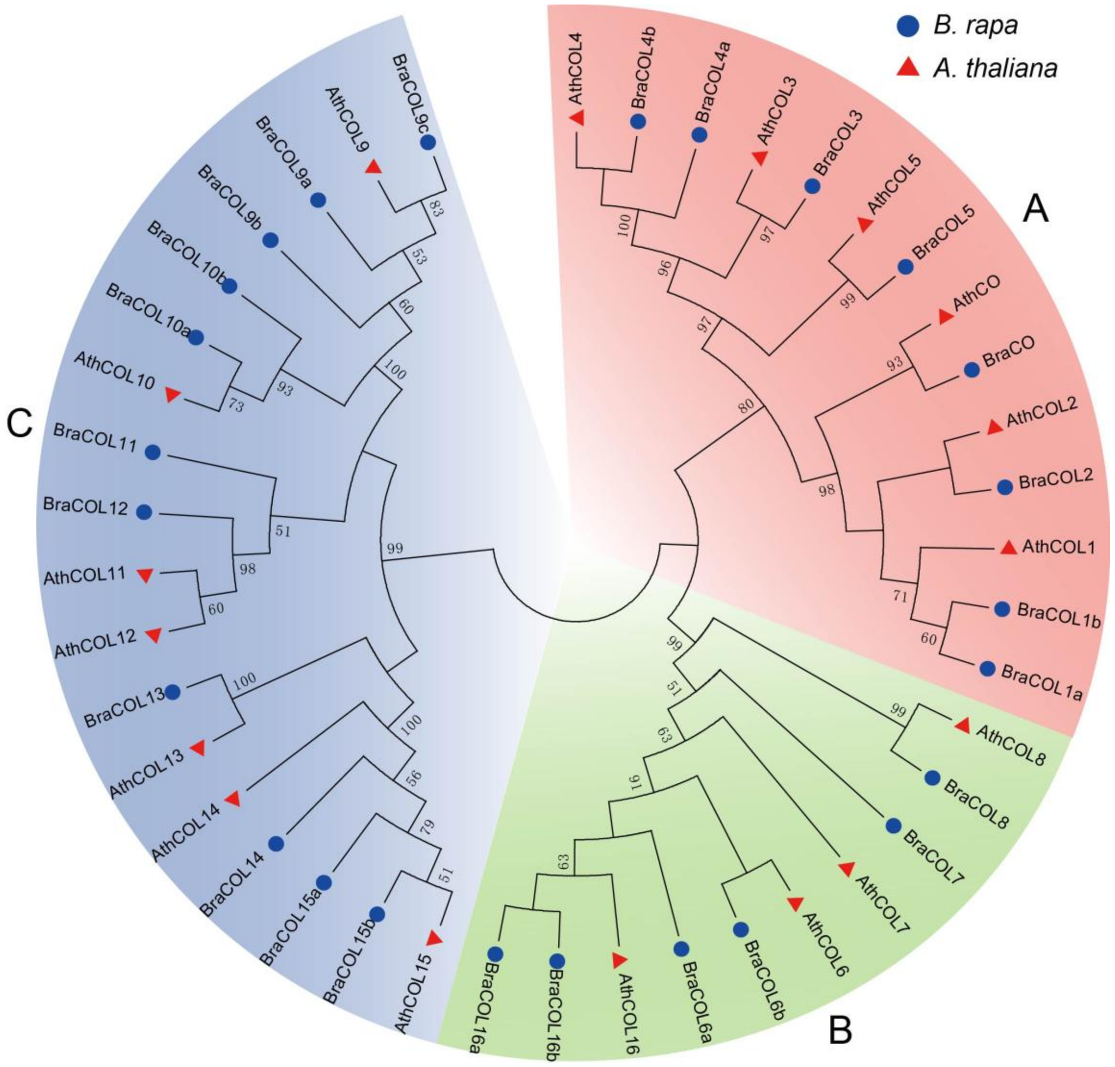

Figure S4. Phylogenetic tree of the *COL* genes in Chinese cabbage and *Arabidopsis* constructed using the neighbour-joining method with MEGA 5. The numbers are the bootstrap values based on 1,000 iterations. Only bootstrap values with >50 % support are indicated.

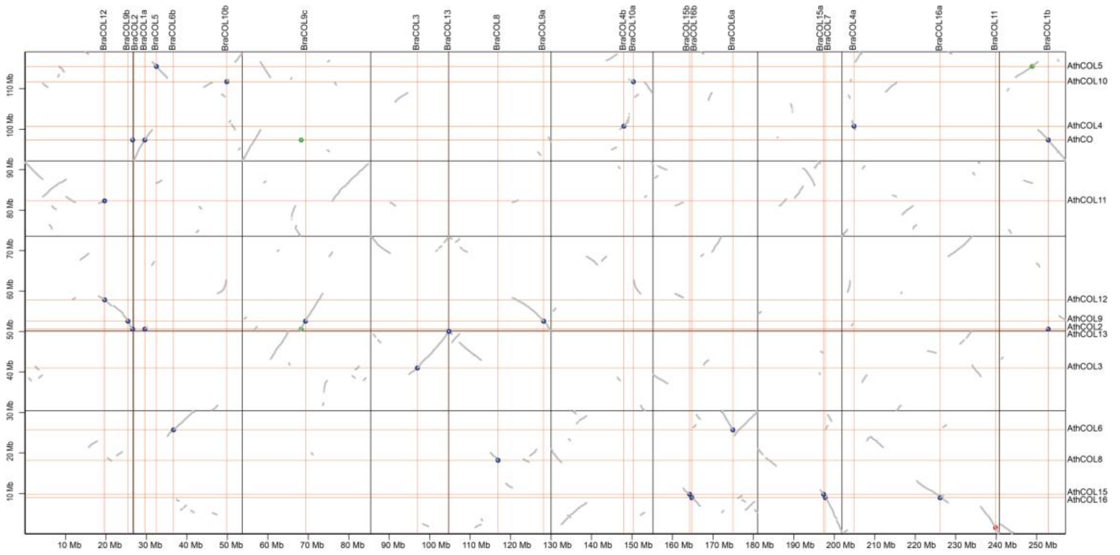

Figure S5. The collinearity of *COL* genes between Chinese cabbage and *Arabidopsis.*

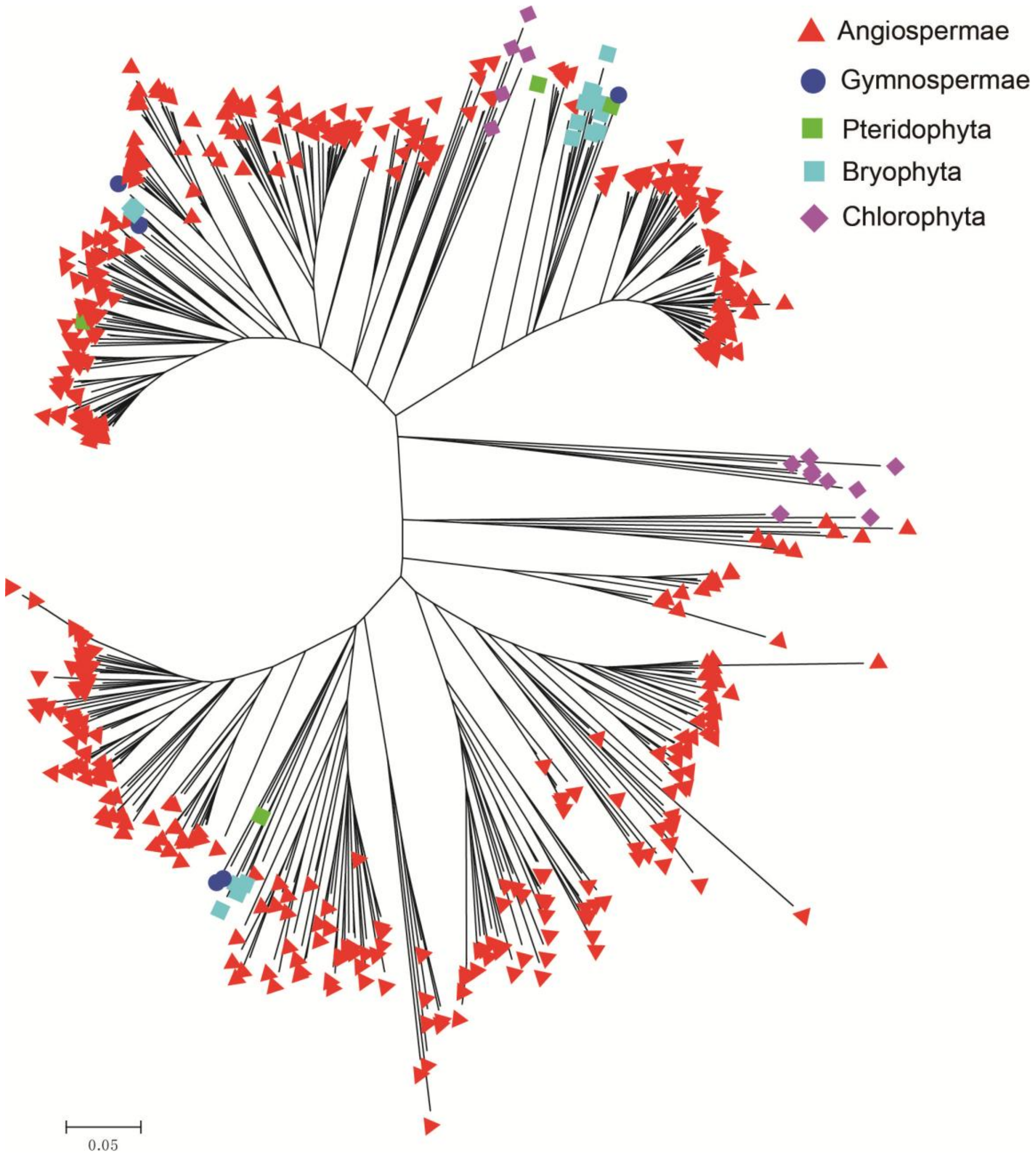

Figure S6. The phylogenetic trees of the *COL* genes from 34 Angiospermae, three Gymnospermae, one Pteridophyta, one Bryophyta and six Chlorophyta species.

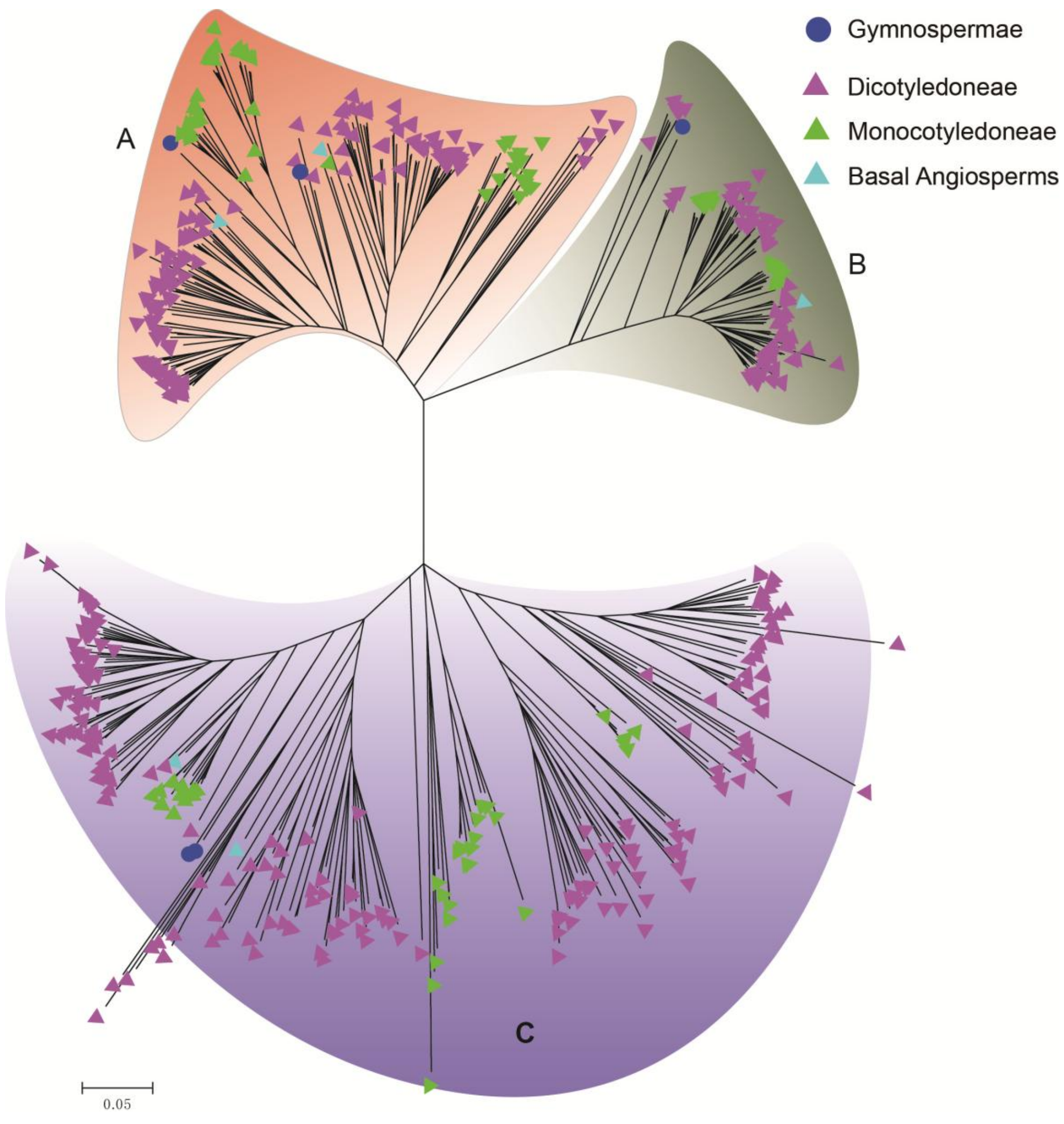

Figure S7. The phylogenetic trees of the *COL* genes from 27 eudicots, six monocots, one basal angiosperm and three Gymnospermae species.

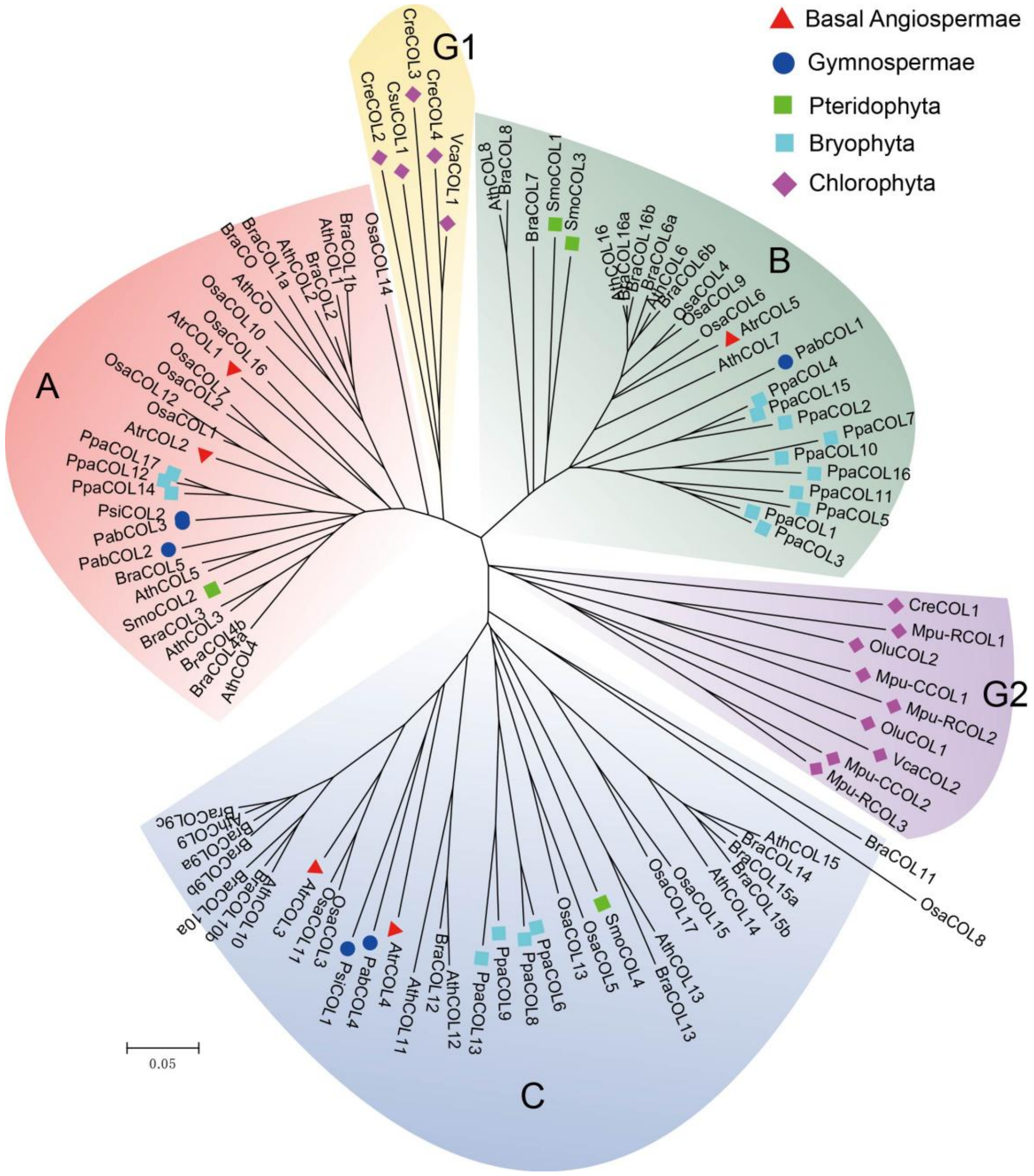

Figure S8. The phylogenetic trees of the *COL* genes from *Arabidopsis*, *B. rapa* and rice with those from three Gymnospermae, one Pteridophyta, one Bryophyta, six Chlorophyta, and one basal Angiospermae species.

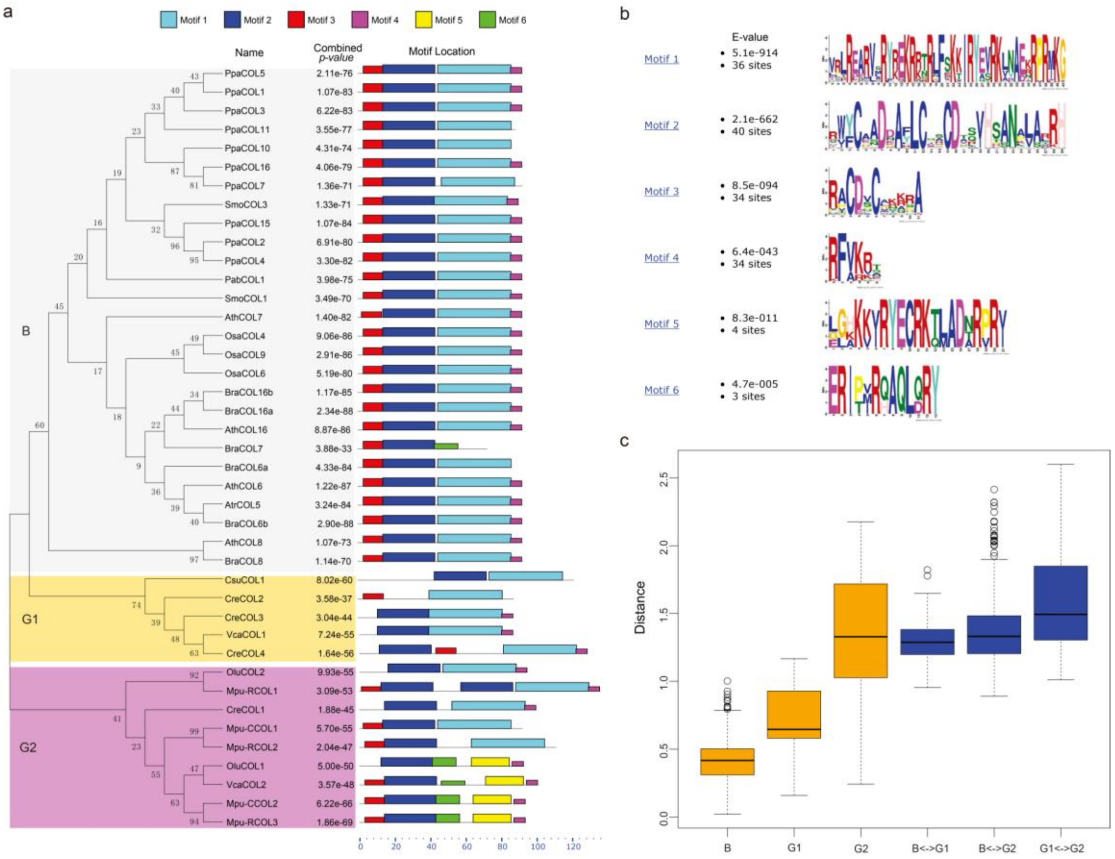

Figure S9. The analysis of the origin of the group B *COL* genes. (a) The phylogenetic tree and six motifs. **(b)** The six best-conserved motif sequences. **(c)** The genetic distance among groups B, G1 and G2.

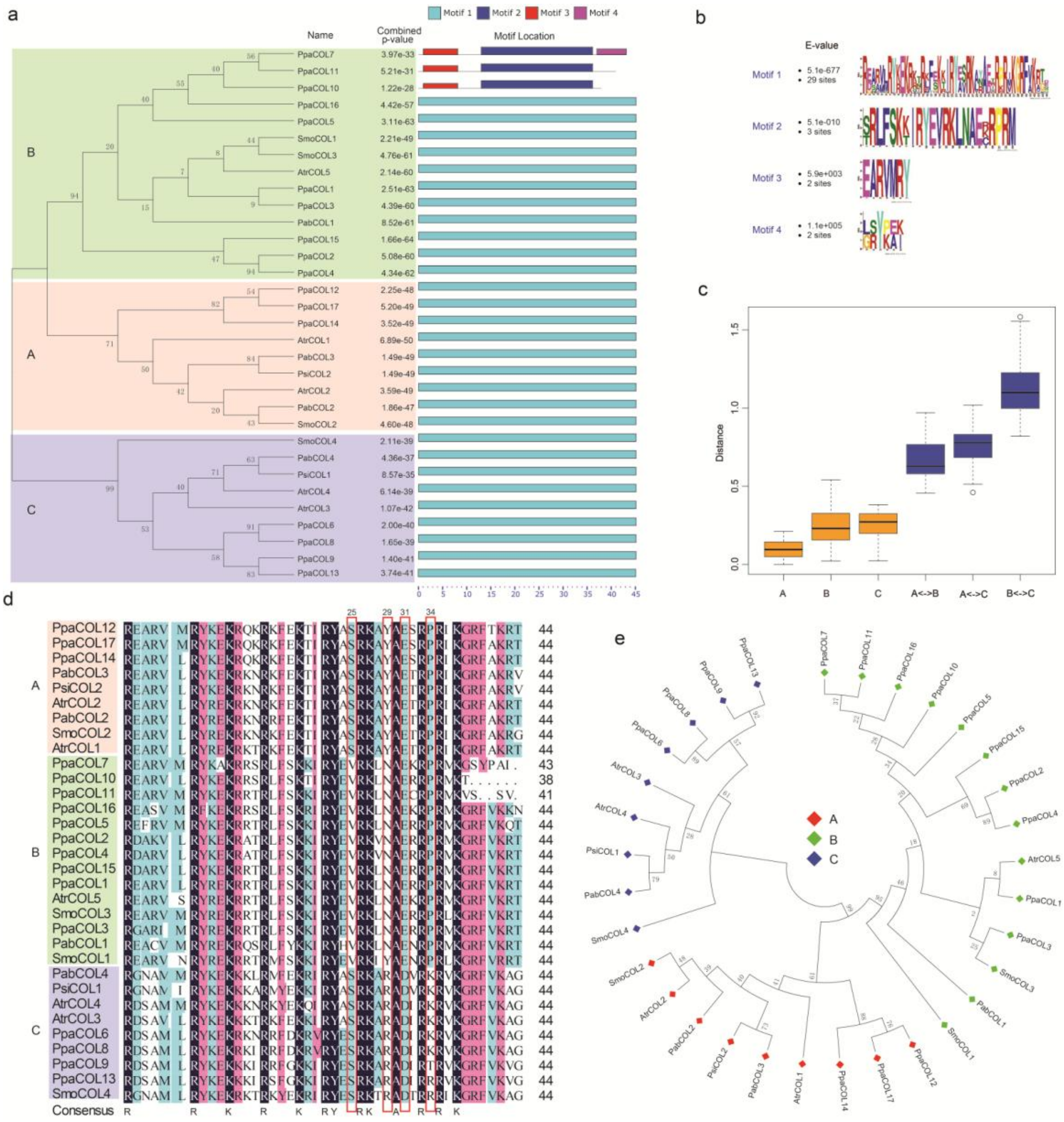

Figure S10. The analysis of the CCT domain in Bryophyta, Pteridophyta, Gymnospermae and basal Angiospermae. (a) The phylogenetic tree and motifs of the CCT sequences in these species. **(b)** The four best-conserved motif sequences. **(c)** The genetic distance among groups A, B and C in these species. **(d)** The alignment of multiple CCT sequences belonging to groups A, B and C. **(e)** The phylogenetic tree of the CCT sequences.

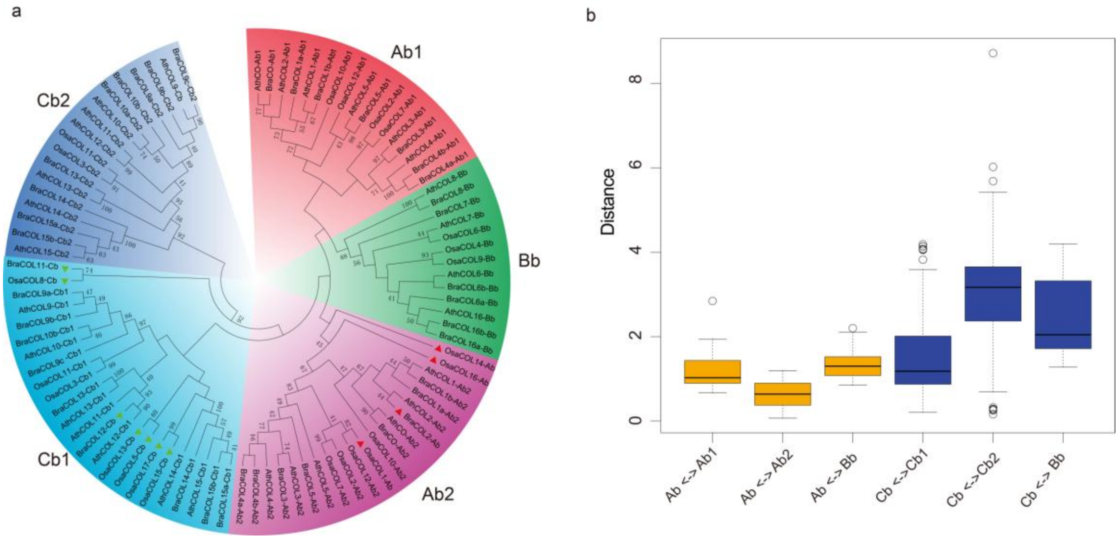

Figure S11. The analysis of the B-box domain lost in *B. rapa*, *Arabidopsis* and rice. Ab1 and Ab2 represent the two B-box domains of group A, Cb1 and Cb2 represent the two B-box domains of group C, and Bb represents the one B-box domain of group B. Ab and Cb represent the B-box domains of group A and group C genes containing only one B-box domain, respectively. **(a)** The phylogenetic tree of the B-box sequences. **(b)** The genetic distance among the B-box sequences.

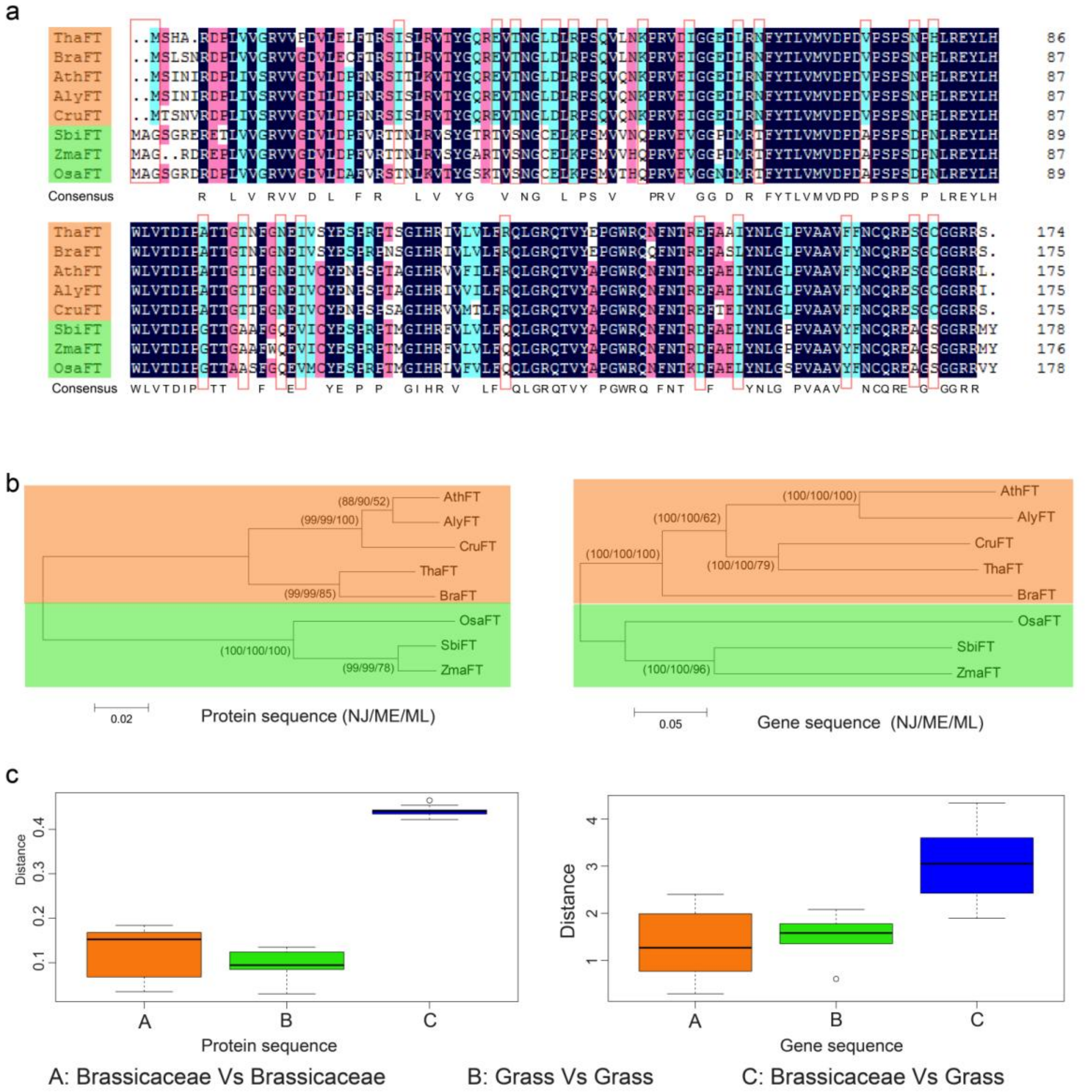

Figure S12. Comparative analysis of *FT* genes between *Brassicaceae* and *Grass* species. (a) The multiple sequence alignment of *FT* genes. The orange solid rectangle represents the five *Brassicaceae* species, and the green solid rectangle represents the three *Grass* species. The red hollow rectangle represents the family-specific amino acids. **(b)** The phylogenetic trees constructed using the protein and gene sequences of *FT*. The numbers on the branches of the phylogenetic tree represent the bootstrap supports provided by the NJ, ME and ML methods. **(c)** The genetic distance in *Brassicaceae* and *Grass* was estimated using the protein and gene sequences of *FT*.

Hydrophilic | Hydrophobic

Species	1	2	3	4	5	6	7	8	9	10	11	12	13	14	15	16	17	18	19	20	21	22
A. thaliana	Ile	Glu	Thr	Leu	Asp	Arg	Gln	Lys	Ile	Leu	Asn	Val	Asn	His	Ala	Thr	Asn	Ile	Arg	Glu	Ile	Phe
B. rapa	Ile	Glu	Thr	Leu	Asp	Arg	Gln	Lys	Ile	Leu	Asn	Val	Asn	His	Ala	Thr	Asn	Ile	Arg	Glu	Ile	Phe
O. sativa	Thr	Thr	Ser	Cys	Glu	Lys	Met	Gln	Val	Met	Thr	Ala	Asp	Asn	Gly	Ala	Gln	Val	Gln	Asp	Leu	Tyr

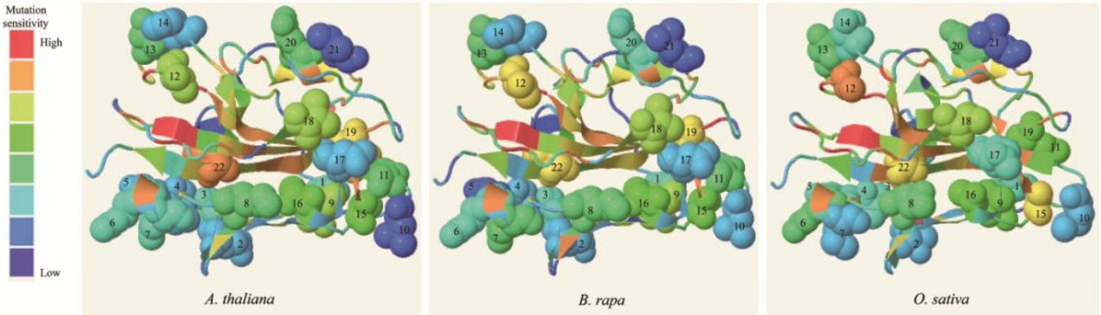

Figure S13. The protein structure of FT predicted for *Arabidopsis*, Chinese cabbage and rice. The 22 family-specific amino acids in FT proteins are marked on the protein structure. The orange solid rectangle represents the hydrophilic amino acids, and the blue solid rectangle indicates the hydrophobic amino acids.

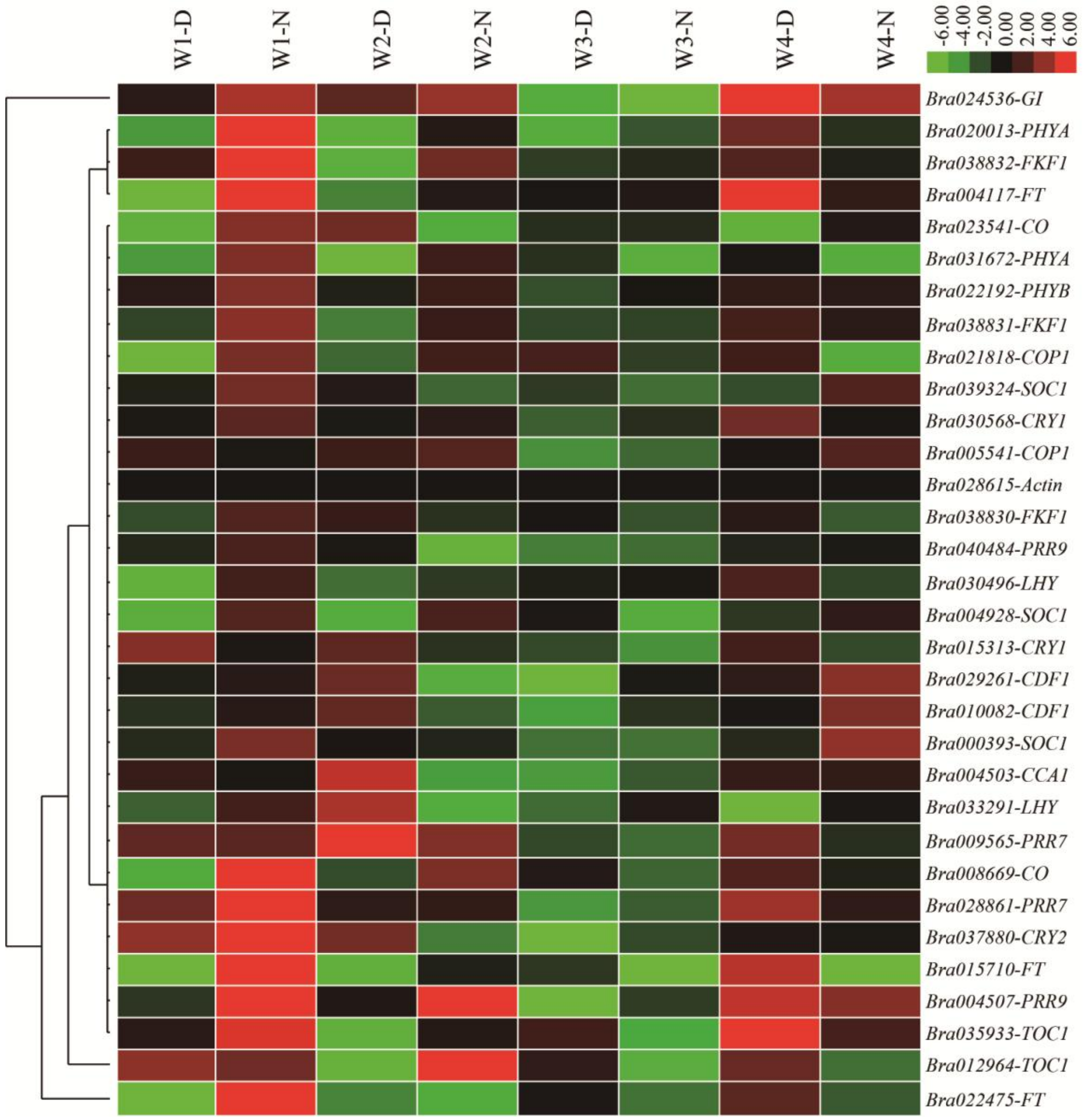

Figure S14. The expression of key photoperiod pathway and circadian clock-related genes under LD and SD conditions for four weeks. W, D and N represent week, day and night, respectively. The RNA expression level relative to the *actin* gene was calculated using the $2^{-\Delta\Delta CT}$ method, and the relative expression under long-day (LD) conditions relative to that under short-day (SD) conditions was calculated and log2-transformed.

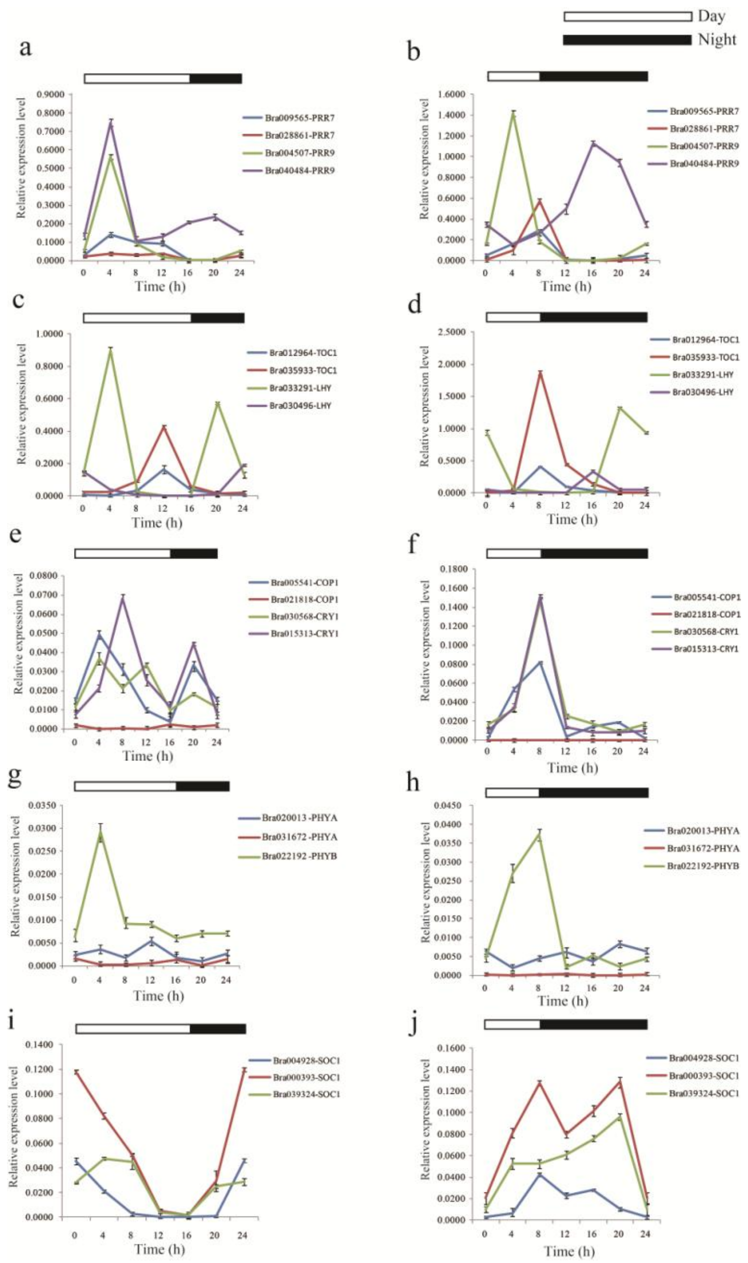

Figure S15. Expression analysis of the photoperiod pathway and circadian clock-related genes by RT-qPCR. The bars correspond to the means ± S.D. from three independent experiments. The relative expression of the (a) *PRR7* and *PRR9* genes, (c) *TOC1* and *LHY* genes, (e) *COP1* and *CRY1* genes, (g) *PHYA* and *PHYB* genes, and (i) *SOC1* genes in Chinese cabbage under LD conditions during a one-day period. The relative expression of the (b) *PRR7* and *PRR9* genes, (d) *TOC1* and *LHY* genes, (f) *COP1* and *CRY1* genes, (h) *PHYA* and *PHYB* genes, and (j) *SOC1* genes in Chinese cabbage under SD conditions during a one-day period.

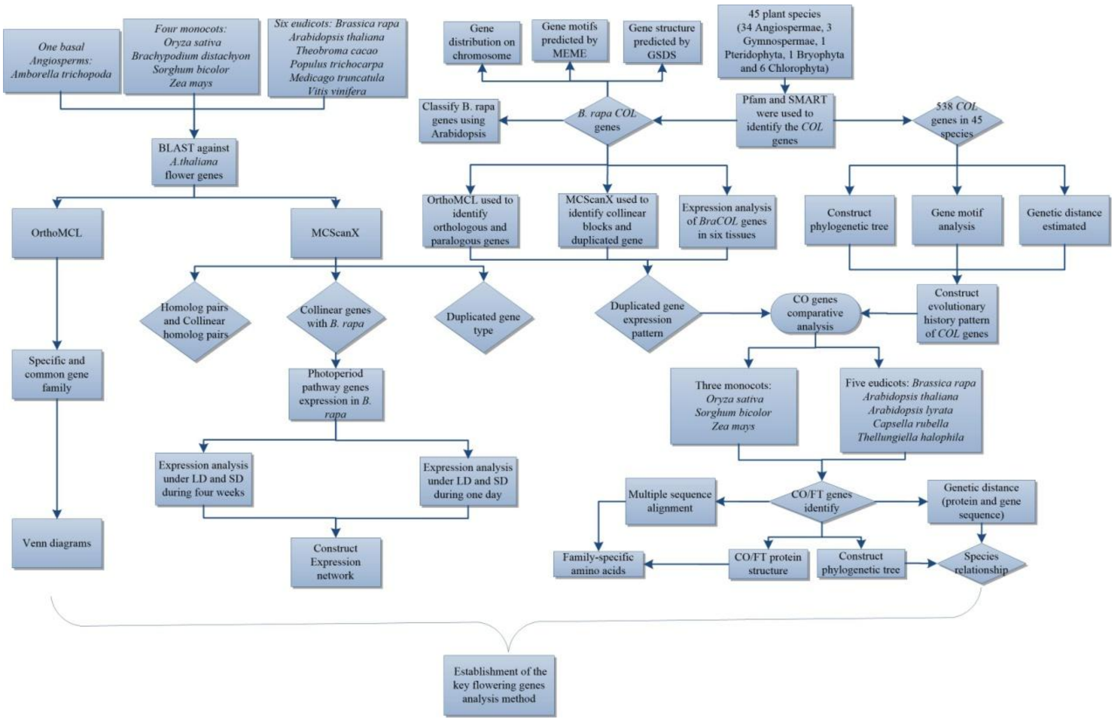

Figure S16. The computational pipeline of the integrated comparative genomic analysis performed in this study.
